# The Impact of Pre- and Postarrival Mechanisms on Self-rated Health and Life Satisfaction Among Refugees in Germany

**DOI:** 10.3389/fsoc.2021.693518

**Published:** 2021-07-06

**Authors:** Elena Ambrosetti, Hans Dietrich, Yuliya Kosyakova, Alexander Patzina

**Affiliations:** ^1^ MEMOTEF Department, Sapienza University of Rome, Rome, Italy; ^2^ Department Education, Training, and Employment Over the Life Course, Institute for Employment Research (IAB), Nuremberg, Germany; ^3^ Department Migration and International Labour Studies, Institute for Employment Research (IAB), Nuremberg, Germany; ^4^ Chair of Sociology, Area Societal Stratification, University of Bamberg, Bamberg, Germany

**Keywords:** refugees, self-rated health, life satisfaction, premigration stress factors, postmigration stress factors, IAB-BAMF-SOEP survey of refugees

## Abstract

In this study, we focus on the evolution of refugees’ well-being in the first years after their arrival in Germany. In contrast to other immigrants (e.g., labor migrants), refugees experience higher risks of unexpected and traumatic events and insecurity before and during their migration and face various legal and structural barriers in the receiving country. We contribute to the existing literature by exploring from a dynamic perspective possible pre- and postarrival determinants of refugees’ life satisfaction and self-rated health upon arrival in Germany and the development of their life satisfaction and self-rated health in the process of becoming established. Applying linear regression and panel models with recent longitudinal data from the IAB-BAMF-SOEP Survey of Refugees in Germany, we find significant effects of prearrival factors, such as traumatic experiences and the complexity of migration, on both life satisfaction and self-rated health at the time of the first interview. Regarding postarrival factors, our results suggest that improvement in language proficiency and labor market status significantly shape refugees’ life satisfaction and self-rated health. The time-dynamic analyses reveal substantial improvements in life satisfaction upon the approval of refugee status and the transition from shared housing to private accommodations. However, we find no improvements in self-rated health due to legal status but rather deterioration effects due to long-term residence in shared housing.

## Introduction

The recent surge in the number of asylum applications in the EU28 member states, which received nearly 5 million first-time applications between 2014 and 2020 (Eurostat, 2021), has raised new and multiple challenges at the national and supranational levels. Germany plays a prominent role in the management of refugee^1^ flows given that many of these asylum applications were submitted there (1.9 Mio of the first-time applications between 2014 and 2020; Eurostat, 2021). Compared to Germany’s previous experience of receiving large inflows of refugees in the early 1990s, the current situation is different in its scale, in part because many recent refugees originate from countries with a limited return perspective, at least in the short term (Degler and Liebig, 2017; Brücker et al., 2020).

Refugees are different from other categories of immigrants because of their traumatic experience (both at home and during migration) and their “forced” decision to migrate (Chiswick et al., 2008; Chin and Cortes, 2015; FitzGerald and Arar, 2018; Kogan and Kalter, 2020). They usually come from destabilized countries, typically affected by wars or war-like situations and human rights violations (UNHCR, 2020). Many refugees take life-threatening routes to arrive at safer destinations. For instance, approximately one-quarter of the recent refugees who arrived in Germany reported shipwrecks, two-fifth reported physical assaults, and 15 percent of female refugees reported sexual assaults (Brücker et al., 2016). After arriving at the destination country, refugees often live under precarious conditions (Robjant et al., 2009; Nickerson et al., 2010); are worried about family members remaining in countries of origin or other transition countries (Nickerson et al., 2010; Löbel and Jacobsen, 2021); have to go through lengthy and, in many cases, stressful asylum procedures; (Silove et al., 1998; Laban et al., 2004; Kosyakova and Brenzel, 2020; Kosyakova and Brücker, 2020), and face restrictions to healthcare services (Silove et al., 1999; Norredam et al., 2006; Chase et al., 2017; Jaschke and Kosyakova, 2021). It has also been argued that – compared to economic immigrants – refugees are less oriented towards the labor market in their migration decision and, hence, are less likely to be positively selected based on health (Chiswick et al., 2008). All of these factors make refugees particularly vulnerable in regard to their health situations: previous studies have found that up to 30 percent of adult refugees suffer from health impairments (Fazel et al., 2005; Robjant et al., 2009; Bakker et al., 2014; Giua, 2017). It is, therefore, not surprising that some scholars have termed the situation of refugees in Germany as a mental health crisis (Schauer, 2016; Hajak et al., 2021).

Given that health status and overall life satisfaction form an important basis for successful and sustainable integration into the economy (Chatterji et al., 2011) and society (Steptoe et al., 2015) of the destination country, it is not surprising that refugees’ integration processes are slower than those of other categories of immigrants (Chiswick et al., 2008; Brücker et al., 2019; Brell et al., 2019). These disadvantages seem to be boosted by additional postmigration stress, such as the length of the procedure to achieve refugee status, different regulations for access to the labor market according to the regulations of the host country, high human capital but difficulties obtaining recognition for their educational degrees and previous work experiences, and lack of knowledge of the host country language (Jacobsen, 2019; Arendt et al., 2020; Kogan and Kalter, 2020; Kosyakova and Brenzel, 2020).

While previous research has focused on refugees’ psychological distress and the prevalence of depression and anxiety symptoms (for meta-analyses and review, *see*, e.g., Fazel et al., 2005; Porter and Haslam, 2005; Lindert et al., 2009; Steel et al., 2009), the self-rated health status and life satisfaction of refugees have been less often addressed by quantitative studies. We contribute to the existing literature by *1*) exploring pre- and postarrival mechanisms affecting refugees’ self-rated health status and life satisfaction upon their arrival in Germany and *2*) the development of their self-rated health and life satisfaction as they become established in Germany. In the social sciences, both self-rated health status and life satisfaction have been shown to effectively predict the risk of mortality (*see* Idler and Benyamini, 2006 for a review), and the examination of these measures has become a conventional way to address the health status of the population of interest (Frijters et al., 2005; Jones and Schurer, 2011; Schmitz, 2011).^2^ These measures reflect slightly different aspects of subjectively perceived health situations and may provide a more comprehensive view of an individual’s overall health status since they link both mental and physical health status.

Using longitudinal data from the IAB-BAMF-SOEP Survey of Refugees in Germany (Brücker et al., 2017) from 2016 to 2019, we study the impact of pre- and postarrival stress factors on the development of self-rated health status and the overall life satisfaction of refugees. We focus on four main groups of refugees arriving in Germany in approximately 2015, namely, refugees from Syria, Iraq, Afghanistan and Eritrea. Including variables beyond sociodemographic individual characteristics such as age, education, social background and gender, the richness of our dataset allows us to account for a variety of pre- and postarrival factors^3^ to address the causes and processes of refugee migration. Adopting a dynamic perspective, our research agenda follows three major questions: *1*) To what extent do different prearrival factors (reasons to migrate, complexity of the migration move, financing of migration and traumatic experiences) affect refugees’ self-rated health and life satisfaction? *2*) What is the contribution of postmigration experiences to refugees’ self-rated health and life satisfaction? *3*) How are changes in refugees’ self-rated health status and life satisfaction shaped by different postarrival experiences?

The handful of studies examining the health status of recent refugees in Germany (Dietrich et al., 2019; Walther et al., 2020; Jaschke and Kosyakova, 2021; Löbel and Jacobsen, 2021) have focused primarily on access to healthcare services and the role of family ties abroad, as well as the role of pre- and postarrival stress for psychological and physical health and psychological distress (*see* meta-analysis by Hajak et al., 2021). These studies, however, mainly focused on the level differences while neglecting the role of the postarrival experiences for the development of health outcomes over the duration of the stay. The theoretical strength of our study is that we test various mechanisms of pre- and post-migration strengths not only from static but also from a dynamic perspective.

## Theoretical Background

### Self-rated Health Status and Life Satisfaction Among Immigrants and Refugees

In the literature on refugees, most studies address well-being by measuring psychopathology, while positive and subjective dimensions of well-being have received significantly less attention (Tozer et al., 2018). Positive well-being is different from mental health, and it accounts for distinct or supplementary conditions considered necessary for an individual to flourish (Keyes, 2007). The subjective components of well-being are receiving growing attention owing to the increasing awareness of the limits of objective indicators in evaluating individual and societal well-being (*see*, e.g., Stiglitz et al., 2009; Bache, 2019). While economists, psychologists and sociologists have studied the determinants of happiness, migration scholars have yet to fully recognize the importance of studying immigrants’ subjective well-being (Wright, 2011; Hendriks and Bartram, 2019). In this study, we address this gap by focusing on two different dimensions of well-being, namely, self-rated health status and overall life satisfaction. In contrast to most of the literature taking a descriptive approach, this paper applies a mechanism-based approach of causal explanation.

Subjective well-being can be used to evaluate immigration processes within the country of residence, as it takes into account immigrants’ perceptions and opinions about their own lives (e.g., Hendriks and Bartram, 2019; Paparusso, 2019). Indeed, subjective well-being is a personal evaluation that people make of their own lives (Amati et al., 2018). Hendriks and Bartram (2019) stressed that the measure of subjective well-being by means of a comprehensive indicator incorporating several life domains has many advantages. This approach does not exclude any domain a priori. Individuals are able to weigh the importance of different aspects of life for themselves and to evaluate their own outcomes. In addition, subjective measures of well-being may be more effective than objective measures of outcomes because they take into account different personal aspirations and expectations. Indeed, individuals with similar levels of well-being as assessed by objective measures may report different outcomes in terms of subjective well-being (Hendriks and Bartram, 2019).

Previous studies addressing the recent inflows of refugees in the German context have identified various health risks that recent refugees suffer from. While these health risks can be partly attributed to past experience, current circumstances in the country of settlement seem to play an important role as well. For instance, Dietrich et al. (2019) and Renner et al. (2020) found symptoms of anxiety, depression, and posttraumatic stress disorder (PTSD) among Syrian refugees living in Germany due to both pre- and postmigration factors. Similar results were obtained by Georgiadou et al. (2018), who found lower levels of mental illness among Syrian refugees than in previous studies, attributing the better health outcomes to postmigration factors, such as living conditions, in Germany. Walther et al. (2020) also found that postmigration factors such as greater stability, secure legal status, nontemporary housing, family reunification, language abilities and social contacts had a positive impact on refugees’ mental health outcomes and subjective life satisfaction (*see* also Gambaro et al., 2018; Löbel and Jacobsen, 2021). Jaschke and Kosyakova (2021) found evidence of significant health improvements in terms of self-related health and well-being among refugees when they were provided early and comprehensive access to the health system.

While addressing various aspects of refugees’ health determinants in general, previous research lacks a longitudinal perspective of the potential role of pre- and postarrival stress factors in various dimensions of well-being. Due to the specific nature of refugees’ migration in which pre- and perimigration factors overlap, we distinguish pre- and postarrival factors, which will be closely discussed in the following subsections.

### The Role of Prearrival Experiences

The literature has shown that refugees indicate poorer health outcomes in general and poorer mental health outcomes in particular than other types of immigrants (Chin and Cortes, 2015). For instance, analyzing studies published between 1966 and 2002, Fazel et al. (2005) found that the prevalence of mental illness among refugees is much more widespread than in the native population. The greater health deprivation of refugees has been partly attributed to the “refugee-producing event that they faced – the persecution or threat of persecution” (Chin and Cortes, 2015, 609).

For instance, in a systematic review of studies conducted between 1990 and 2007, Lindert et al. (2009) found that refugees are at higher risk of depression than labor migrants. Indeed, contrary to labor migrants, refugees have been exposed to **traumatic experiences** such as violence and political repression in their home countries. Similar results were obtained by Kirmayer et al. (2011), who reviewed papers published between 1998 and 2009 on the mental health of migrants and refugees. They found that refugees who have had severe exposure to violence often have higher rates of trauma-related disorders, including posttraumatic stress disorder and chronic pain or other somatic syndromes (*see* also Schweitzer et al., 2011). Steel et al. (2009) found that refugees exposed to torture and political repression are at higher risk of mental illness.

Moreover, Schapendonk et al. (2020) conducted a broad literature review on the complexity, fragility and indetermination of refugees’ **migration pathways**. The role of migration pathways was also addressed by Beiser and Hou (2016), who explored the role of perimigration encounters by examining the time spent in refugee camps. This negative experience during the migration process was positively associated with refugees’ emotional problems but not with aggressive behavior. In the case of complex migration pathways, the distinction between migration from the home country or the country of last residence challenges the distinction between pre- and perimigration. Paniagua et al. (2021) identified the search for well-being as both a causal driver of the pathway of migration and an outcome of migration.

Although economic stress factors do not constitute major reasons for refugee migration (Chiswick, 1999; Chin and Cortes, 2015), several prior studies have stressed **financial stress** before migration as shaping refugees’ health outcomes. For instance, Kim et al. (2020) reported strong associations between financial stress and depression in African refugees in the United States. Dietrich et al. (2019) found significant effects of financing refugee moves on young Syrian and Iraqi PTSD diagnoses. A possible explanation here is that refugees with accumulated financial resources have better opportunities to decrease the risks and negative expectations associated with refugee migration. In turn, Bauer et al. (2020) showed reduced life satisfaction for refugees previously living in higher classes in their countries of origin. This finding was explained in terms of the stronger risks of downward mobility that higher classes face when arriving in the receiving country (Bauer et al., 2020).

### The Role of Postarrival Experiences

In addition to prearrival stressors, the literature on refugees’ health outcomes and subjective well-being stresses the importance of postmigration experiences (Kirmayer et al., 2011; Bogic et al., 2015; Li et al., 2016). According to the well-known “healthy migrant effect” (Antecol and Bedard, 2006; Kennedy et al., 2015), in the first period after arrival in receiving countries, migrants report better health than the native population. Indeed, migrants are positively self-selected from the population of their countries of origin (Borjas, 1987). However, a large number of studies have shown that the initial advantage of migrants decreases over time and generations (Mladovsky, 2011) and with the changing composition of migrant inflows (Kristiansen et al., 2016). Several factors can account for the loss of health advantages of migrants: early life conditions in the country of origin play an important role; nevertheless, exposure to risk in the destination country also plays a prominent role. Migrants are often exposed to several kinds of deprivations, both material and immaterial, such as poverty, social exclusion, poor housing, discrimination, and social isolation, leading to the so-called “exhausted migrant effect” (Bollini and Siem, 1995; Nazroo, 2003; Borrell et al., 2015; Cela and Barbiano di Belgiojoso, 2019). These stressful experiences place migrants’ health at risk: migrants often work and live in poor environments, lack protective factors such as close family members, and lack or have limited access to health care, exposing them to risky behaviors such as smoking and alcohol abuse, unhealthy diets and sedentary lifestyles (Antecol and Bedard, 2006; De Luca et al., 2013; Borrell et al., 2015; Kristiansen et al., 2016).

Some recent studies have argued that the stress that refugees experience in destination countries (a.k.a. postmigration stress) may heighten their existing mental problems (e.g., Porter and Haslam, 2005; Li et al., 2016) and be an even more important predictor of refugees’ mental health status than traumatic experiences before and during the flight (but, *see* Schweitzer et al., 2011; e.g., Beiser and Hou, 2016). In the following, we focus on several postmigration stressors that have been identified as important determinants of refugees’ health outcomes.

The first postarrival stress factor is the acquisition of an established **legal status**. For instance, Kosyakova and Brenzel (2020) demonstrated the relevance of the timing of the granting of legal status regarding individuals’ access to education and the labor market (*see* also van Tubergen, 2010; Hainmueller et al., 2016; Hvidtfeldt et al., 2019). Likewise, Robjant et al. (2009) revealed a negative impact of living in detention centers on refugees’ health, which is likely related to uncertainty about future residence status, fear about deportation and precarious living conditions in the facilities. In this sense, lengthy asylum procedures may directly and indirectly trigger and exacerbate the trauma that refugees have suffered in their countries of origin or during flight (Coffey et al., 2010). Silove et al. (1998) identified the asylum process, e.g., regular contact with public authorities, as a stressor itself in the setting of Tamil refugees in Australia. This observation is consistent with the findings of Laban et al. (2004), who provided evidence for the adverse effects of lengthy asylum procedures on the mental health of Iraqi refugees in the Dutch context.

A second postarrival stress factor relates to the **housing situation**. A lack of access to stable and secure housing has been shown to heighten refugees’ stress levels (Porter and Haslam, 2005; Georgiadou et al., 2018; Walther et al., 2020; Jaschke and Kosyakova, 2021) since life in temporary collective accommodations hinders privacy and autonomy and increases isolation from the local community (Adam et al., 2019). Moreover, poor housing situations might be linked to the financial and social deprivation that refugees are likely to experience in the early arrival stages (Krahn et al., 2000).

The research has also identified that **labor market** access – which refugees often lack – is a key factor affecting refugees’ health and well-being. In this context, unemployment has been identified as an important predictor of mental health problems (Kim, 2016) and postmigration depression among refugees (Beiser and Hou, 2001). These findings were further supported by Maqul et al. (2020), who stressed the role of structural integration in refugees’ life satisfaction. Similarly, a meta-analysis by Porter and Haslam (2005) indicated an increase in mental health indicators associated with economic prosperity after migration.

A fourth factor is the acquisition of the **language of the receiving country**. Previous studies have implied that the lack of host country language fluency is a significant predictor of depression in the postmigration period (e.g., Beiser and Hou, 2001; Söndergaard and Theorell, 2004; Kartal et al., 2018). Indeed, refugees who are not fluent in the host country language experience a lack of integration both in society and in the labor market. For instance, Green (2017) and Aljadeeah et al. (2021) documented the importance of language knowledge and the existence of language barriers among refugees in Germany, which prevented them from having full access to health care (*see* also Jaschke and Kosyakova, 2021).

## Data and Method

### IAB-BAMF-SOEP Survey of Refugees in Germany

For our analysis, we rely on data from the IAB-BAMF-SOEP Survey of Refugees in Germany, a longitudinal survey of refugees and their household members conducted annually (Brücker et al., 2017). The target population for this survey is drawn from the Central Register of Foreigners (*Ausländerzentralregister*, AZR), the national registry of all foreign citizens in Germany. The survey covers all individuals seeking asylum or any other form of protection, irrespective of their current legal status, who arrived in Germany for humanitarian reasons between 2013 and 2016 and were registered in the AZR by January 2017.

The first wave of the survey was conducted between June and December 2016 and included 4,465 adult refugees (i.e., aged above 17 in the interview year). The gross participation rate was approximately 50 percent of addresses originally drawn, which is substantially higher than the participation rate of comparable surveys of the German population (Kroh et al., 2017). Interviews were conducted face-to-face with computer assistance (CAPI) and were supported by interpreters, if needed. The questionnaires were available in seven languages (Arabic, English, Farsi/Dari, German, Kurmanji, Pashtu, and Urdu) and included auditory instruments for survey participants who were illiterate. The second wave included 67 percent of the participants in the first wave as well as an additional sample, resulting in the collection of data from 2,559 panel respondents and 2,897 first-time respondents (Brücker et al., 2020). The response rate of the panel respondents in the third wave was 68 percent, and the panel stability was 80 percent (Britzke and Schupp, 2020). The fourth-wave response rate amounted to 65 percent, and the panel stability was 89 percent (The SOEP Group, 2020). As a result, the data from the IAB-BAMF-SOEP Survey of Refugees in Germany included 8,321 adult persons (18 yr and older) who contributed 18,342 person-year observations over the four survey waves.

### Analytical Sample

For our analyses, we restrict the initial sample to refugees from Afghanistan, Eritrea, Iraq and Syria (1,490 respondents were dropped). This restriction ensures that we consider the quantitatively largest refugee groups that arrived in the time window of 2013–2016. We also exclude individuals who were identified as nonrefugees (59 respondents were dropped) and those above age of 55 at the first interview (273 respondents were dropped). Further, we confine our sample to refugees who arrived between 2014 and 2016 in Germany to ensure duration of stay of a maximum of 3 yr before the fourth interview (545 respondents were dropped). For similar reasons, we keep only respondents with their first interview taking place maximum 3 yr after arrival in Germany (196 respondents were dropped). Given the dynamic lens of our analyses, we further restrict our data to respondents with a first interview in 2016 or 2017 (359 respondents were dropped) and those who had participated in at least 2 survey waves (1,425 respondents were dropped). Finally, we drop respondents with missing information on dependent variables in the first interview (17 respondents were dropped). In total, this approach yields an unbalanced panel including 3,957 individuals with 11,464 observations. Supplementary Table S1 explains the sample selection in more detail.

### Dependent Variables

We consider self-rated health and life satisfaction as dependent variables. For the analyses of self-rated health, we rely on the question “How would you describe your current state of health?” Respondents could answer on a scale ranging from 1 (“poor”) to 5 (“very well”). This question is a widely used item in many health studies in the social sciences. Research on this particular question has shown that self-assessment is a strong predictor of mortality because it proxies general physical well-being (e.g. Mossey and Shapiro, 1982). In general, self-rated health is argued to combine “the subjective experience of acute and chronic, fatal and nonfatal diseases, and general feelings of well-being, such as feeling run down and tired or having backaches and headaches” (Mirowsky and Ross, 2008, 104). Therefore, the employed self-assessment also incorporates some mental health aspects. In general, however, it approximates the physical health domain.

In contrast, life satisfaction represents the cognitive dimension of individuals’ lives. We rely on the well-established 11-point scale that is used in many long-running panel surveys around the world and that is widely used by researchers (Lucas, 2007; Green, 2011). Empirically, we rely on answers to the question “How satisfied are you currently with your life in general?” Respondents could answer on a scale ranging from 0 (“totally dissatisfied”) to 10 (“totally satisfied”). The life satisfaction construct refers to “the degree to which an individual judges the overall quality of his/her own life as-a-whole favourably. In other words: how much one likes the life one leads” (Veenhoven, 2012, 67). In line with research on self-rated health and mortality, other research has shown that cognitive evaluations of individuals’ lives can also predict mortality (Diener and Chan, 2011). Thus, both outcomes under study constitute outcomes relevant to the quality of individuals’ lives. Furthermore, the employed data empirically support this conjecture and show that the correlation between the two variables amounts to only 0.26 at the first interview and to 0.23 on average over the four observed survey waves.

### Analytical Strategy

We investigate the role of pre- and postarrival factors in refugees’ life satisfaction and self-rated health in four analytical steps.

Based on ordinary least squares (OLS) regressions, we first provide a multivariate description of our dataset to compare the findings for our analytical sample to previous findings in the literature on refugees. In doing so, we investigate the association of important socioeconomic characteristics with the employed outcome measures at the time of the first interview (for details on the variables, *see* Section 3.5).

Second, we investigate the importance of prearrival factors for life satisfaction and self-rated health at the first interview. In doing so, we model the influence of four mechanisms (i.e., reasons for leaving, migration route, financing, and traumatic events) separately, holding the set of control variables constant before estimating a saturated model that accounts for all prearrival mechanisms under study.

Third, we employ fixed effects (FE) estimators to elaborate on the influence of postarrival mechanisms (i.e., changes in legal status, housing situation, German language proficiency, and work status) on the outcome measures under study. The advantage of employing FE models is that time-constant unobserved heterogeneity no longer biases the estimates (Allison, 2009). Compared to estimators which rely on between person variation and therefore on the unit homogeneity assumption, FE models rely on the temporal homogeneity assumption (Firebaugh et al., 2013). As we are explicitly interested in how changes in the migration process are associated with refugees’ life satisfaction and health, FE estimations that rely on a weaker exogeneity assumption constitute the best way to describe this process.

Fourth, we take a dynamic perspective and investigate how changes in postarrival statuses affect changes in life satisfaction and self-rated health over time with random effects growth curve (REGC) models.^4^ As we are particularly interested in whether transitions to certain postarrival statuses lead to convergence or divergence of life satisfaction or health profiles over time, we employ an estimator relying on between person variation. For this purpose, we rearrange the coding of the postarrival variables and always analyze three different groups. We show the development of the life satisfaction and self-rated health of refugees who had already been granted protection status at the first interview and contrast their well-being development with that of refugees who were granted protection status later or who were never granted protection status during the observation period. The four postarrival states under study are the possession of a permanent work contract, the possession of protection status, improvement of German language skills, and the housing situation. Employing this view with our longitudinal dataset facilitates the investigation of whether adverse states after arriving in Germany, such as a lack of protection status, impair life satisfaction and self-rated health in the long run and whether transitions out of these adverse states increase both health outcomes.

### Independent Variables

For analysis, we employ a set of explanatory and additional control variables. Regarding explanatory variables, we distinguish between prearrival and postarrival variables, i.e., individuals’ characteristics that developed before or after arriving in Germany. Supplementary Tables S2–S4 in the supplementary online appendix provide descriptive statistics on the prearrival, postarrival and control variables, respectively.

Regarding prearrival stress factors, we consider reasons for leaving the home country, multiple means of transport to reach Germany, migration financing and traumatic events during migration. To capture the multiple motivations for leaving the home country, we employ a count variable, which captures two dimensions of migration motives that go beyond political reasons such as persecution and fleeing because of war and conflict:^5^ social reasons (referring to friends and family) and economic reasons. Regarding migration financing, we distinguish between financial support from family or friends and financing through marketing assets (an individual’s own properties or labor). To capture the burden of migration, we code a count variable indicating the possible use of multiple means to move from the country of origin to Germany. This variable reflects both the duration and complexity of the move in a straightforward way. The variable trauma events reflects the number of a variety of traumatic experiences, which are typically recognized as causal factors for PTSD (Silove et al., 1997).

As introduced in Section 3.4, we also explore the role of socioeconomic characteristics of refugees in their well-being outcomes. The considered socioeconomic factors include age, gender, premigration educational attainment, perceived premigration socioeconomic status (relative to that of others in the origin country), the location of the partner at the first interview and German language proficiency. Furthermore, we describe the influence of legal status and accommodation type at the first interview and show the importance of accounting for methodological factors.

While prearrival variables are retrospectively measured and time constant, postarrival variables may vary over waves. As time-varying postarrival stress factors, we employ protection status, labor market status, housing situation, and German language proficiency. We distinguish among three forms of legal status that could characterize refugees in Germany: *1*) protection status is still under approval, *2*) protection is granted, and *3*) protection status is denied. Over time, the “protection is granted” status becomes the absorbing status. The variable housing situation distinguishes among individuals living in *1*) shared housing and *2*) private houses or flats. The acquisition of German language proficiency ranges from very poor to very good on a 5-point scale and reflects the respondents’ self-assessment of their German skills. Labor market status indicates individuals’ position at the time of interview and follows the ILO concept of labor market status (Brandolini et al., 2006). We distinguish five labor market statuses: *1*) permanent work contract, *2*) temporary work contract (including marginal jobs), *3*) participation in education or training, *4*) job-seeking and *5*) inactivity (including all other activities).

For the dynamic analysis with REGC models, we slightly rearrange these variables and always distinguish among individuals who were already in the “positive” state at the first interview, who transitioned to this “positive” state later, and who never transitioned to the “positive” state (refer to Section 3.4). Furthermore, the dynamic modeling uses time since the first interview dummies as growth factors.

As time-varying control variables, we employ the year of the interview and dummy variables indicating the number of survey waves in which the individuals participated. In doing so, we control both for period effects and panel conditioning (Warren and Halpern-Manners, 2012). The interviews were performed between 2016 and 2019, and the respondents participated in a minimum of two and a maximum of four survey waves. We control for the quality of interviews by means of an indicator for third persons being present at interview (e.g., partner or third persons such as an interpreter or others) and an indicator of whether the respondents were answering sensitive questions. We also account for the respondent’s region of residence (federal state) at the time of the interview.

## Results

### Life Satisfaction and Self-rated Health of Different Refugee Groups

Forced migration typically indicates both adverse conditions in the home country, which motivate migration, and the logistical details of the migration itself. In the first step, we address the association of the socioeconomic situations of refugees who arrived in Germany between 2014 and 2016 and their life satisfaction and self-rated health at the first interview. The corresponding results for life satisfaction and self-rated health are presented in Table 1. For both outcomes, we present a baseline model (Models 1.1 and 2.1) and an extended model (Models 1.2 and 2.2).

**TABLE 1:** Life satisfaction and self-rated health at the first interview.

	Life satisfaction		Self-rated health	
	Model 1.1 (Baseline)	Model 1.2 (Extended)	Model 2.1 (Baseline)	Model 2.2 (Extended)
Country of origin (ref. Syria)				
Afghanistan	0.336**	0.395***	−0.137**	−0.126*
Eritrea	0.364*	0.598***	0.119	0.221**
Iraq	0.207+	0.236*	−0.109*	−0.078
**Sociodemographics**				
Age	−0.042	−0.062*	−0.008	0.001
Age squared	0.000	0.001+	−0.000*	−0.000*
Male		−0.091		0.294***
Highest professional educational attainment (ref. No professional education)				
Vocational education		−0.219		0.034
University		−0.545***		0.042
Other		0.047		0.169
Socioeconomic status (ref. Low)				
Medium		0.165+		0.112**
High		−0.013		0.127**
German language proficiency (ref. None at all)				
Not very good		0.104		0.101+
Average		0.447***		0.231***
Good		0.403*		0.375***
Very good		0.921**		0.563***
Residence of partner (ref. No partner)				
Germany		0.430***		0.036
Abroad		−0.273+		−0.032
**Migration-related factors**				
Arrival year (ref. 2016)				
2014	0.054	−0.280+	0.053	−0.099
2015	−0.050	−0.123	0.111*	0.025
Legal status (ref. In process)				
Protection granted	0.546***	0.403***	0.126**	0.093*
Protection denied	0.119	0.077	0.020	0.010
Type of residence (ref. Shared accommodation/other)				
Private flat/house		0.723***		0.054
**Methodological factors**				
Survey wave at first interview (ref. 2016)				
2017	0.018	−0.064	−0.042	−0.069+
Third person present at the interview (ref. Partner)				
Other third person	−0.660***	−0.354**	−0.096*	−0.119*
No third person	−0.590***	−0.272**	−0.088*	−0.162***
Partner and other third person	−0.359+	−0.343+	−0.099	−0.11
Constant	8.243***	7.607***	4.616***	4.024***
Number of persons	3,957	3,957	3,957	3,957
Adj. *R* ^2^	0.026	0.067	0.094	0.130
F Statistic	4.426	6.951	14.187	13.349

Statistical significance at: +p < 0.10, *p < 0.05, **p < 0.01, ***p < 0.001. OLS regression coefficients. Dependent variables: Overall life satisfaction (0–10) and self-rated health (1–5). Control variables: federal state fixed effects, other legal status and indicators for missing values in independent variables. *Data*: IAB-BAMF-SOEP Survey of Refugees, waves 1–4.

Following the baseline specification, life satisfaction varies significantly by country of origin: compared to refugees from Syria, those from Iraq, Eritrea and Afghanistan report significantly higher life satisfaction (Model 1.1). In line with the literature (Frijters and Beatton, 2012), life satisfaction decreases with age. Note, however, that this relationship becomes statistically significant only when accounting for all model covariates (Model 1.2). Life satisfaction does not vary significantly by year of arrival or by year of the first interview (Model 1.1).

The results from the extended model, which additionally accounts for sociodemographic variables, imply that social origin is significantly related to life satisfaction (Model 1.2). In particular, refugees with university education report lower levels of life satisfaction, while those with medium perceived premigration socioeconomic status report higher levels. The level of fluency in the German language has a positive correlation with life satisfaction. Refugees with a partner living in Germany report higher life satisfaction than those without a partner (singles), while having partners living abroad is negatively related to life satisfaction (Stahnke and Cooley, 2020). In regard to legal status, compared to pending protection, granted protection status is positively related to life satisfaction, while denied protection is not significantly different. Similarly, compared to living in shared or other type of accommodations, living in private housing is positively related to life satisfaction.

In contrast to the results for life satisfaction, those for self-rated health show more variance between countries of origin (Model 1.2). Compared to refugees from Syria, those from Iraq and Afghanistan seem to be worse off, while refugees from Eritrea report better health status in the extended model (Model 2.2). Surprisingly, age is less associated with health status, which might reflect refugees’ specific migration motives and migration experience. In contrast to the results for life satisfaction, we observe a positive correlation between a higher premigration socioeconomic status and self-rated health status. In turns, education is not significantly related to health. With respect to further socioeconomic factors, we find no impact of housing or partnership on self-rated health status and only a weak impact of socioeconomic status. The results for legal status and German language proficiency are similar to those for life satisfaction.

### The Impact of Prearrival Experiences on Refugees’ Life Satisfaction and Subjective Health

In Table 2, we introduce a set of possible prearrival mechanisms affecting life satisfaction (Panel A) and subjective health (Panel B) to the extended models (Model 1.2 and Model 2.2, respectively). We assume that worse premigration circumstances and migration conditions affect refugees’ life satisfaction and self-rated health at the first interview. In particular, we address four possible prearrival mechanisms: reasons for leaving the country of origin, complexity of the move, financing of the move and traumatic experiences while migrating to Germany. In a full model, we test for collinearity of the single mechanism of interest.

**TABLE 2:** Associations between prearrival experiences and life satisfaction and self-rated health at the first interview.

Panel A: LIFE SATISFACTION					
	Model 1.3	Model 1.4	Model 1.5	Model 1.6	Model 1.7
Reasons for leaving (ref. Social reasons only)					
None of these	−0.350*				−0.381*
Economic reasons only	−0.427**				−0.416**
Economic and social reasons	−0.428*				−0.410*
Number of different modes of transport		−0.077**			−0.051+
Financing of migration via family/friends			−0.13		−0.066
Financing of migration via marketing assets			−0.066		0.025
Number of traumatic events				−0.161***	−0.144***
Number of persons	3,957	3,957	3,957	3,957	3,957
Adj. *R* ^2^	0.069	0.07	0.068	0.073	0.074
F Statistic	6.546	6.823	6.551	7.097	6.252
**Panel B: SELF-RATED HEALTH**					
	**Model 2.3**	**Model 2.4**	**Model 2.5**	**Model 2.6**	**Model 2.7**
Reasons for leaving (ref. Social reasons only)					
None of these	−0.092				−0.102
Economic reasons only	−0.147*				−0.139+
Economic and social reasons	−0.133+				−0.119
Number of different modes of transport		−0.015			0.003
Financing of migration via family/friends			−0.083+		−0.047
Financing of migration via marketing assets			−0.009		0.036
Number of traumatic events				−0.106***	−0.105***
Number of persons	3,957	3,957	3,957	3,957	3,957
Adj. *R* ^2^	0.131	0.131	0.131	0.14	0.141
F Statistic	12.281	12.681	12.485	13.654	11.798

Statistical significance at: +p < 0.10, *p < 0.05, **p < 0.01, ***p < 0.001. OLS regression coefficients. Constant not shown. Full regression results in Supplementary Table S5. Dependent variables: Overall life satisfaction (0–10) and self-rated health (1–5). Control variables: country of origin, age; gender; survey year fixed effects; federal state fixed effects; third person present at interview; year of arrival fixed effects; premigration education; legal status at the first interview; type of accommodation; premigration socioeconomic status; location of partner; German language proficiency; and indicators for missing values in independent variables. *Data*: IAB-BAMF-SOEP Survey of Refugees, waves 1–4.

Respondents with multiple motives for migration (social and/or economic motives) generally report lower levels of life satisfaction at the first interview than those oriented by one migration motive (Model 1.3). Moreover, we observe that those reported only social reasons (such as leaving because of family or friends) for leaving country of origin display higher life satisfaction levels compared to other groups. This might reflect the fact that joining the family or social contacts in the destination country is an important driver of refugees’ life satisfaction. This findings have been also mirrored in the literature stressing the positive role of family reunification of refugees’ mental health (Löbel, 2020; Löbel and Jacobsen, 2021).

In the same way, the complexity of the migration move approximated by the number of means of transport negatively affects individuals’ life satisfaction (Model 1.4). For instance, one additional transport mode reduces life satisfaction by 0.08 points. Given that the reported modes of transports vary between 1 and 9, the maximum number of which associates with 0.69 points decrease in life satisfaction. Additional analyses imply that an increasing number of means of transport is correlated with factors such as the type of migration route taken from the country of origin to Germany, temporary residence in third countries and migration duration. Thus, the number of means of transport captures the complexity and stress of migration to Germany.

In turn, we find no correlation of financing migration through marketing assets or social networks on the respondents’ life satisfaction at the first interview (Model 1.5). However, there is a negative impact of traumatic experiences on life satisfaction (Model 1.6). The maximum number of the surveyed traumatic events amounted to seven. With one traumatic event costing 0.161 points on satisfaction scale from 0 to 10, the maximum number of traumatic experiences that the respondents were exposed during migration would reduce refugees’ life satisfaction by more than one point which is substantial.

The full model confirms the single mechanism-based models and indicates no problems of collinearity (Model 1.7). The direction of each mechanism affecting individual life satisfaction supported by the single-mechanism models holds in the full model. The finding that premigration stress factors seem to be important predictors of refugees’ life satisfaction in the first interview indicates that the refugees were still at the beginning of a possible process of integration or adaption to German society at that time.

Regarding self-rated health, the analysis provides only partial support for our mechanism of interest. Multiple motivations for migrations show a weak negative impact on self-rated health at the first interview (Model 2.3). We find no impact of either the complexity of the move (Model 2.4) or the mode of financing the migration (Model 2.5). Nevertheless, we find that multiple exposures to traumatic experiences severely reduce self-rated health (Model 2.5). On the scale between 1 and 5, an exposure to a one traumatic event during migration reduces self-rated health by 0.106 points. Again, the full model confirms the single-mechanism models, but in this model, the impact of multiple motives for migration is weakened (Model 2.6). Thus, refugees’ self-rated health at an early stage of integration into German society seems to be mainly driven by traumatic experience before arrival at Germany.

### The Impact of Postarrival Experience on Refugees’ Life Satisfaction and Self-rated Health

Table 3 depicts the partial correlations of postarrival experiences with life satisfaction (Panel A) and self-rated health (Panel B). As in the previous subsection, we introduce the mechanisms of interest separately before estimating a full model incorporating all mechanisms at once.

**TABLE 3:** Associations between postarrival experiences and life satisfaction and self-rated health.

Panel A: LIFE SATISFACTION					
	Model 3.1	Model 3.2	Model 3.3	Model 3.4	Model 3.5
Legal status (ref. In process)					
Protection granted	0.128				0.095
Protection denied	−0.112				−0.125
Labor market status (ref. Inactive)					
Permanent work contract		0.159+			0.140+
Temporary work contract		0.142			0.127
In education		0.07			0.056
Job seeking		−0.055			−0.069
German language proficiency (ref. None at all)					
Not very good			0.096		0.092
Average			0.236+		0.216+
Good			0.355**		0.316*
Very good			0.501**		0.468**
Type of residence (ref. Shared accommodation/other)					
Private flat/house				0.478***	0.454***
Person-years	11,464	11,464	11,464	11,464	11,464
Number of persons	3,957	3,957	3,957	3,957	3,957
*R* ^2^ overall	0.002	0.002	0.002	0.006	0.009
*R* ^2^ within	0.009	0.01	0.01	0.014	0.018
**Panel B: SELF-RATED HEALTH**					
	**Model 4.1**	**Model 4.2**	**Model 4.3**	**Model 4.4**	**Model 4.5**
Legal status (ref. In process)					
Protection granted	0.085*				0.083*
Protection denied	0.175*				0.168*
Labor market status (ref. Inactive)					
Permanent work contract		0.112**			0.102**
Temporary work contract		0.081*			0.076+
In education		0.060+			0.054
Job seeking		0.060*			0.053+
German language proficiency (ref. None at all)					
Not very good			0.018		0.021
Average			0.076		0.069
Good			0.134*		0.123+
Very good			0.195**		0.184*
Type of residence (ref. Shared accommodation/other)					
Private flat/house				0.043	0.032
Person-years	11,464	11,464	11,464	11,464	11,464
Number of persons	3,957	3,957	3,957	3,957	3,957
*R* ^2^ overall	0.029	0.03	0.032	0.023	0.044
*R* ^2^ within	0.009	0.009	0.01	0.008	0.012

Statistical significance at: +p < 0.10, *p < 0.05, **p < 0.01, ***p < 0.001. FE regression coefficients. Constant not shown. Full regression results in Supplementary Table S6. Dependent variables: overall life satisfaction (0–10) and self-rated health (1–5). Control variables: gender, age, survey year at first interview, federal state fixed effects, third person present at the interview, dummies indicating the number of interviews (panel conditioning), and indicators for missing values in independent variables. *Data*: IAB-BAMF-SOEP Survey of Refugees, waves 1–4.

Model 3.1 tests whether changes in legal status shape refugees’ life satisfaction. Receiving a protection status appears to be positively associated with life satisfaction, while denials of applications lead to lower life satisfaction; however, the results do not reach the conventional level of statistical significance. Model 3.2 shows the role of labor market transitions in changes in life satisfaction. The results imply that changes from inactive to work or education appear to be positively associated with changes in life satisfaction. The partial correlation between life satisfaction and permanent work seems to be mostly pronounced. In turn, changing from an inactive work status to a job-seeking work status slightly decreases life satisfaction, albeit not statistically significant. The results further imply that with increasing German language skills over time, life satisfaction increases (Model 3.3). In particular, improving German language skills to a good or a very good level (from not at all) is associated with increases in life satisfaction of 0.355 and 0.501 points, respectively. Model 3.4 further shows that a change in residence from shared or other accommodations to a private flat or house leads to an increase in life satisfaction by 0.478. The full model suggests no changes in the direction and almost no changes in the size of the coefficients when all four mechanisms of interest are included simultaneously (Model 3.5). Accordingly, the mechanisms under study have independent influences on life satisfaction.

Regarding self-rated health, Model 4.1 shows that receiving protection status is positively associated with self-rated health: the change from an unclear legal situation to granted protection exhibits a statistically significant partial correlation of 0.09. At the same time, we also observe that the change to a rejected application is positively and significantly related to self-rated health. It appears that it is not a type of decision but having a decision on asylum application which is important for self-rated health. Similar relationship was established for refugees’ labor market entry and language course entry (Kosyakova and Brenzel, 2020), supporting the idea that waiting in limbo is particularly detrimental for refugees’ health outcomes (Bakker et al., 2014). The second postarrival mechanism related to refugees’ labor market situation implies that all transitions – transition to permanent working contract in particularly – out of inactive work status are positively and statistically significantly associated with increases in self-rated health (Model 4.2). Similar to the findings on life satisfaction, improvements in German language skills over time are positively associated with changes in self-rated health (Model 4.3). However, only the substantial improvement in German skills from no skills at all to good or very good German language proficiency is significantly different from zero and increases self-rated health by 0.13 and 0.20 points, respectively. In contrast to the findings on life satisfaction, a change from shared accommodations to a private flat or house is not statistically significantly associated with increases in self-rated health (Model 4.4). The inclusion of all mechanisms in Model 4.5 again suggests no changes in direction and almost no changes in the size of the coefficients, implying independent influences of the mechanisms under study on self-rated health.

### Development of Refugees’ Life Satisfaction and Self-rated Health Over Time and the Role of Postarrival Experiences

**FIGURE 1:**
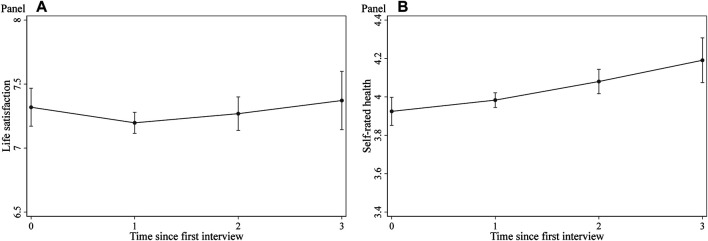
Development of life satisfaction and self-rated health over time. *Note*. Conditional profile plots estimated by random effects growth curve models of life satisfaction (left-hand side) and self-rated health (right-hand side). The full regression results are displayed in Supplementary Table S7 (model S7.1) and S8 (model S8.1) of the online appendix. Dependent variables: Overall life satisfaction (0–10) and self-rated health (1–5). Control variables: dynamic postarrival mechanisms (labor market status, legal status, language proficiency, and accommodation), gender, age, socioeconomic status before migration, arrival year, highest professional educational attainment, survey year fixed effects, federal state fixed effects, third person present at the interview, dummies indicating the number of interviews (panel conditioning), indicator for answering sensitive questions, and indicators for missing values in independent variables. *Data*: IAB-BAMF-SOEP Survey of Refugees, waves 1–4.

To explore the overall development of refugees’ well-being, Figure 1 depicts the changes in life satisfaction (left-hand side) and self-rated health (right-hand side) since the first interview. While the data exhibit only small overall changes in life satisfaction (with the overall development following a slight u-shaped pattern), self-rated health increases over time.

**FIGURE 2:**
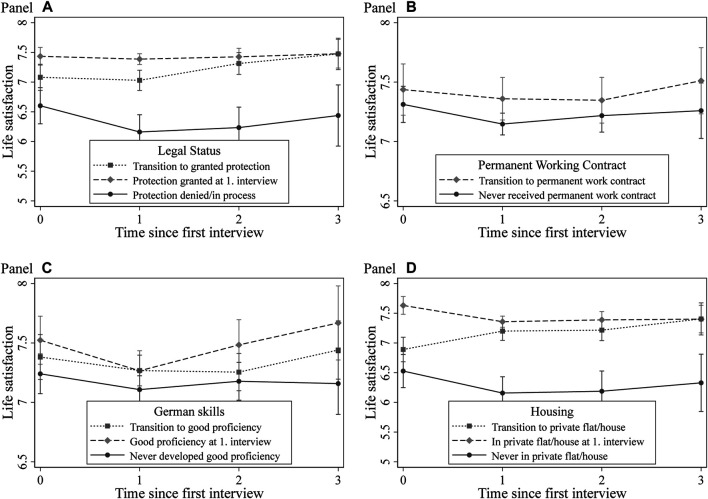
Development of life satisfaction by postarrival mechanism. *Note*. Conditional profile plots of the influence of postarrival mechanisms on life satisfaction estimated by random effects growth curve models. The full regression results are displayed in Supplementary Table S7 (models S7.2 to S7.5). Dependent variables: overall life satisfaction (0–10). Control variables: gender, age, socioeconomic status before migration, arrival year, highest professional educational attainment, survey year fixed effects, federal state fixed effects, third person present at the interview, dummies indicating the number of interviews (panel conditioning), indicator for answering sensitive questions, and indicators for missing values in independent variables. *Data*: IAB-BAMF-SOEP Survey of Refugees, waves 1–4.

**FIGURE 3:**
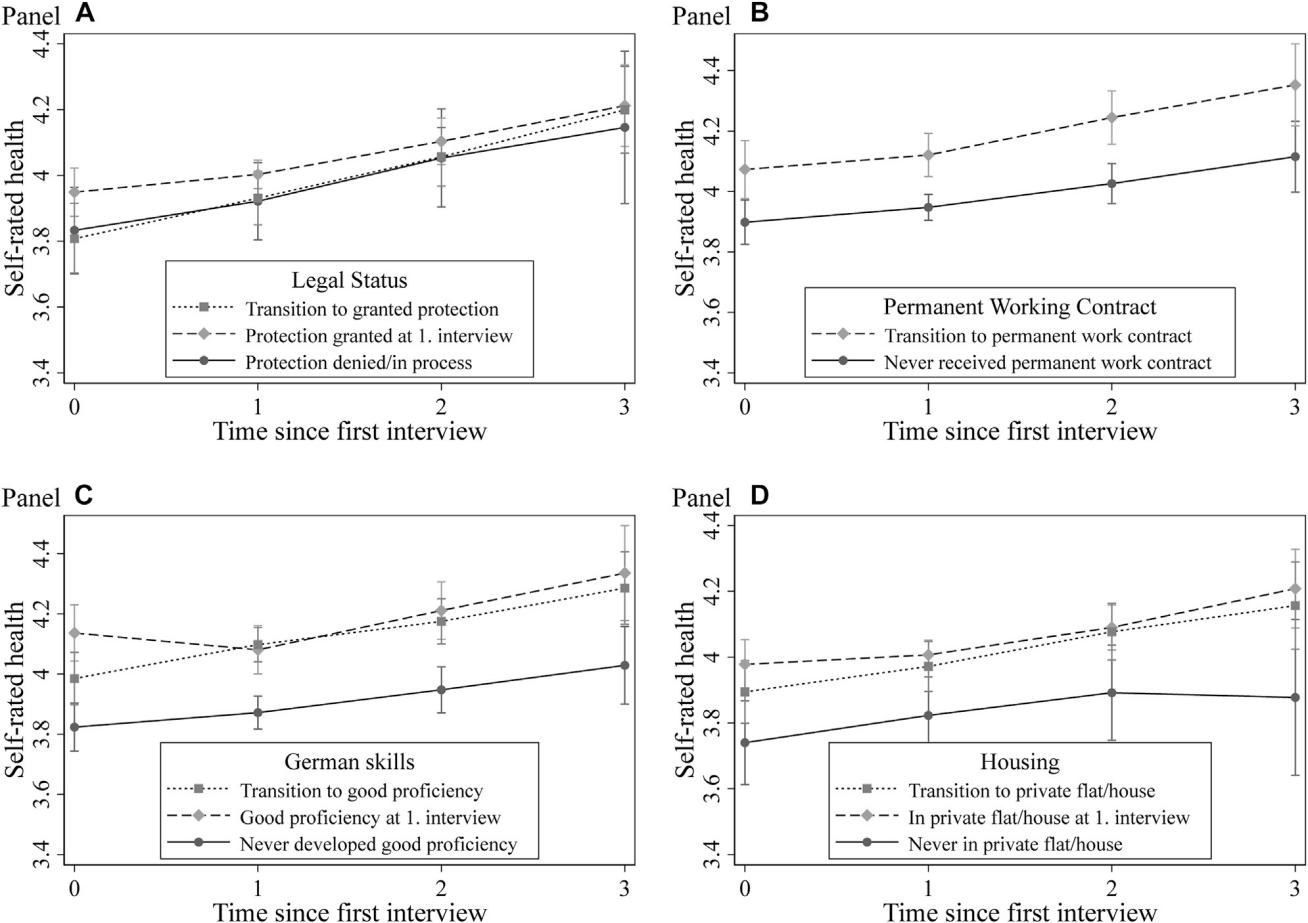
Development of self-rated health by postarrival mechanism. *Note*. Conditional profile plots of postarrival mechanisms on self-rated health by random effects growth curve models. Full regression results in Supplementary Table S8 (model S8.2 to S8.5). Dependent variables: Self-rated health (1–5). Control variables: gender, age, socioeconomic status before migration, arrival year, highest professional educational attainment, survey year fixed effects, federal state fixed effects, third person present at the interview, dummies indicating the number of interviews (panel conditioning), indicator for answering sensitive questions, and indicators for missing values in independent variables. *Data*: IAB-BAMF-SOEP Survey of Refugees, waves 1–4.

To examine in detail the importance of changes in postmigration experiences for changes in refugees’ life satisfaction and self-rated health over time, we slightly rearrange the coding of the postarrival variables regarding postmigration experiences (refer to Section 3.4 and Section 3.5). In our REGC models, we now distinguish between refugees who never switch to a certain state and refugees who have a “positive” postmigration experience. Figures 2, 3 depict the results from the corresponding empirical exercise on refugees’ life satisfaction (Figure 2) and self-rated health (Figure 3). For the full models, see supplementary online appendix Supplementary Tables S7, S8.

Panel A in Figure 2 illustrates the development of life satisfaction by legal status. In particular, we plot the development of life satisfaction for *1*) refugees with granted protection at the first interview, *2*) those who transitioned from waiting for a decision to receiving protection status, and *3*) those who never received protection over the observation window. The results clearly show that refugees with granted protection at the first interview initially have overall higher life satisfaction and that their life satisfaction changes slightly over time. For the group of refugees who receive protection later during the observation window, we observe first a slight decrease and then an increase in life satisfaction levels. For this group, we also observe a remarkable convergence of their life satisfaction levels with those of refugees with initially granted protection. In turn, refugees with denied protection show a decreasing trend in life satisfaction over the observation period. Overall, these results reveal that granted protection leads to increased life satisfaction.

To examine the role of labor market transitions, Panel B in Figure 2 presents the development of life satisfaction for refugees who transition from nonpermanent employment, inactivity or other statuses to permanent employment and those who never make such a transition. Since the group of refugees who already had permanent employment at the first interview is very small, the estimations for this group are imprecise and, therefore, not displayed in Figure 3. The general trend is that transitions to permanent work result in higher life satisfaction levels in the long run. We also observe cumulative effects of language skills on the development of life satisfaction over time (Panel C in Figure 2). While life satisfaction of refugees with poor language skills declines over time, it increases among refugees with good or very good German skills at the first interview. This finding might indicate that skills in the language of the destination country are a crucial precondition for integration in the receiving society – a finding in line with the prior literature on refugees (van Tubergen, 2010; Arendt et al., 2020).

Turning to the housing situation, the results in Panel D in Figure 2 reveal a substantial impact of housing conditions on the development of refugees’ life satisfaction over time. In part, staying in shared accommodations during the entire observation period leads to a one-point lower life satisfaction level than that of refugees in private flats or houses at the time of the first interview. In turn, the life satisfaction levels of refugees transitioning from shared accommodation to private flats or houses converge with those of groups who initially had better housing situations.

In the final step, we replicate these models for refugees’ self-rated health status; Figure 3 illustrates the results. These dynamic analyses reveal slightly different conclusions than those for refugees’ life satisfaction. First, we find no differences in the development of self-rated health by legal status (Panel A in Figure 3), thereby confirming the findings from our FE models presented in Table 3 (Panel B). Regarding the labor market situation, the results suggest a positive impact of transitions to permanent work on self-rated health (Panel B in Figure 3). After 3 yr, differences between the two groups becomes even more pronounced, suggesting a long-lasting positive effect of structural integration on refugees’ reported health status. Panel C in Figure 3 shows the results for the association between German skills and the development of self-rated health. The results indicate that improvements in language skills are not associated with long-lasting increases in health levels and that refugees with poor language skills throughout the observation period have the lowest levels of self-rated health. This result provides a hint for a possible selection effect. Likewise, changes in housing conditions do not seem to play a significant role in the development of self-rated health over time (Panel D in Figure 3).

## Discussion

The unprecedented inflow of refugees in Germany since 2015 has raised multiple challenges in terms of their integration into society. In this context, studying refugees’ well-being outcomes is crucial because health status and overall life satisfaction are fundamental in shaping successful and sustainable integration into the economy (Chatterji et al., 2011) and society (Steptoe et al., 2015) of the destination country. Compared to other (labor) migrants, refugees’ migration processes are typically more abrupt and often accompanied by threatening events due to wars, oppression, discrimination and violation of their human rights before and during their flights (Chiswick, 1999; Hatton, 2020; UNHCR, 2020). Moreover, refugees typically face substandard conditions in the places they live after they enter the host country and suffer from additional postmigration stress, such as lengthy asylum procedures, family reunification, financial burdens or cultural integration (Adam et al., 2019; Dietrich et al., 2019; Brücker et al., 2020; Walther et al., 2020; Jaschke and Kosyakova, 2021). It is not surprising, therefore, that refugees face worse well-being outcomes and greater health risks than types of other migrants (Fazel et al., 2005; Robjant et al., 2009), resulting in a slower integration process for them (Brücker et al., 2019).

In this study, we examined the impact of pre- and postarrival stress factors on the development of the subjective well-being (overall life satisfaction and self-rated health) of four main groups of refugees arriving in Germany in approximately 2015, namely, refugees from Syria, Iraq, Afghanistan and Eritrea. Empirically, we relied on the most recent longitudinal data from the IAB-BAMF-SOEP Survey of Refugees (2016–2019) representative for the recent refugee population in Germany and panel analysis techniques. In contrast to previous studies on recent refugees’ health and well-being outcomes conducted in the German context, we do not only consider the level differences but also explicitly employed a dynamic perspective.

Overall, our results suggest that refugees’ life satisfaction and, to a lesser extent, their self-rated health at an early stage of integration into German society are mainly driven by complexity of the move and particularly traumatic experiences before arrival at Germany. However, from a longitudinal perspective, we also provide evidence that both studied well-being outcomes improved slightly over the observation period. Accordingly, our results indicate a marginal overall recovery process of the arriving population. These findings are in clear contrast to the “healthy migrant narrative” and the related postarrival assimilation pattern (Antecol and Bedard, 2006; Kennedy et al., 2015). Indeed, previous research findings suggest that refugees do not fall within the healthy migrant paradox because they experience higher levels of mental and physical health problems than the general population in the host country (Fazel et al., 2005; Gerritsen et al., 2006a).

Our results confirm the findings of previous research on refugees’ self-rated health and well-being. Consistent with a well-established literature in this field (e.g., Chin and Cortes, 2015; Dietrich et al., 2019; Walther et al., 2020), immediately after their arrival in Germany, refugees’ life satisfaction and self-rated health are negatively affected by premigration stressors, such as multiple reasons for leaving the country of origin, the complexity of the migration route and especially the extent of traumatic events, while financial burdens show no effects on either well-being outcome. Regarding postarrival mechanisms, our research findings suggest that integration into German society measured by labor market participation, German language acquisition, legal status and independent housing plays an important role in improving the life satisfaction of refugees. In addition, progress towards integration measured by legal status, labor market participation and language acquisition have a positive impact on self-rated health, while the effect of independent housing is less strong. Similar results were reported in a large strand of literature focusing on the role of postmigration factors in shaping the life satisfaction and self-rated health of the refugee population. Previous studies have stressed integration policies in host countries as a fundamental factor in fostering the well-being of refugees (e.g., Tip et al., 2019; Walther et al., 2020; Jaschke and Kosyakova, 2021). Correspondingly, such policies should not neglect the impact of past traumatic experiences on refugees’ health and well-being; thus, health care services in host countries should be designed to meet the needs of these populations (Gerritsen et al., 2006b; Mölsä et al., 2014; Jaschke and Kosyakova, 2021). In addition, our dynamic models indicate improvements in self-rated health and life satisfaction with decreases in refugees’ postarrival stress factors by means of improved language proficiency or economic integration. Accordingly, policymakers should direct their efforts towards policies that are particularly productive for fostering refugees’ integration processes into the labor market and society of the destination country. Finally, our findings raise questions about the lasting effects in the self-rated health and well-being of refugees of the policies implemented to foster independent housing, labor market incorporation, and language acquisition. Future research should address the role of social networks in mediating the tapering-off of the positive effects of these interventions. Nevertheless, further evaluations are necessary to support causal interpretation of the observed association.

Our study did not examine more homogenous subgroups, for instance, by gender or education. Additionally, we have to consider that the refugees addressed in this study came from rather heterogeneous country-specific and cultural backgrounds. In addition to differences regarding the origin of the refugees, we must admit that the variance within the key explanatory variables was limited. On the one hand, the granting of legal status, at least for the Syrian group, occurred mostly in advance of the first interview, while integration into high-level education (such as apprenticeship training or academic studies) or significant jobs occurred at moderate levels even 4 yr after the refugees’ arrival. These transitional steps seem to require a longer setup time, as expected at the beginning of the period of refugee influx into Germany.

## Data Availability

The data analyzed in this study is subject to the following licenses/restrictions: This article uses the factually anonymous data of the IAB-BAMF-SOEP Survey of Refugees, waves 1–4. The IAB-BAMF-SOEP Survey of Refugees in Germany is a representative longitudinal survey conducted jointly by the Institute for Employment Research (IAB) in Nuremberg, the Research Centre on Migration, Integration, and Asylum of the Federal Office for Migration and Refugees (BAMF-FZ) and the German Socio-Economic Panel (SOEP) at the DIW Berlin. Data access was provided *via* a Scientific Use File supplied by the Research Data Centre (FDZ) of the German Federal Employment Agency (BA) at the Institute for Employment Research (IAB). DOI: 10.5684/soep.iab-bamf-soep-mig.2019. All documentation concerning the IAB-BAMF-SOEP Survey of Refugees and including questionnaires and data manuals are made available by the FDZ (https://fdz.iab.de/en/FDZ_Individual_Data/iab-bamf-soep.aspx) and DIW (https://www.diw.de/sixcms/detail.php?id=diw_01.c.814095.en). Due to the German Data Protection legislation, we cannot make the original data from the IAB-BAMF-SOEP Survey of Refugees or the dataset we generated available. Researchers can however apply for data access *via* the FDZ or DIW. The computer code for the analysis is available at https://osf.io/tc3sn/. Requests to access these datasets should be directed to https://fdz.iab.de/en/FDZ_Individual_Data/iab-bamf-soep.aspx.

## Footnotes

1 With the use of the term “refugee,” we refer to individuals who seek asylum outside their countries of origin or any other form of protection, irrespective of their legal status (Brücker et al., 2020, 26).

2 There is, however, some criticism of this approach in the literature due to the differences in the self-rated assessment of health across cultures (Jylha et al., 1998) and differences in reference points (Ubel et al., 2003).

3 In response to the complexity of many recent refugees’ escape paths, we combine pre- and perimigration stressors (Walther et al., 2020) as prearrival factors and contrast them with postarrival factors. Due to the structure of our data, prearrival information was collected at the first interview and thus was already partially overshadowed by early postarrival experiences, including approval of the legal status of refugees in Germany, for a subgroup of respondents.

4 Results from hierarchical growth curve models are identical (refer to Supplementary Figures S1, S2 in supplementary online appendix).

5 Following the IAB-BAMF-SOEP Survey of Refugees, 97 percent of the respondents reported having left their countries of origin due to war or civil war, persecution, discrimination or forced labor in our sample. For these reasons, we do not consider political reasons in our measure for multiple motivations for leaving the home country.
