# Supplementary material for: The Impact of Pre- and Postarrival Mechanisms on Self-rated Health and Life Satisfaction Among Refugees in Germany
Source: Front Sociol. 2021 Jul 6;6:693518. doi: 10.3389/fsoc.2021.693518 (PMC8289889; doi:10.3389/fsoc.2021.693518)
Supplement: Supplementary file 1 [file Table1.DOCX]

Supplementary Material

**Figure S1**Development of life satisfaction over time

**

*Note.* Conditional profile plots estimated by a linear random effects growth curve model of life satisfaction as presented in the manuscript (left-hand side) and hierarchical random effects growth curve model of life satisfaction (right-hand side). Dependent variables: Overall life satisfaction (0 to 10). Control variables: dynamic postarrival mechanisms (labor market status, legal status, language proficiency, and accommodation), gender, age, socioeconomic status before migration, arrival year, highest professional educational attainment, survey year fixed effects, federal state fixed effects, indicator for third person present at the interview, indicator for no one else except the interviewer present at the interview, dummies indicating the number of interviews (panel conditioning), and indicator for answering sensitive questions*.*

*Data:* IAB-BAMF-SOEP Survey of Refugees, waves 1 to 4*.*

**Figure S2:** Development of self-rated health over time

**

*Note*. Conditional profile plots estimated by a linear random effects growth curve model of self-rated health as presented in the manuscript (left-hand side) and hierarchical random effects growth curve model of self-rated health (right-hand side). Dependent variables: Self-rated health (1 to 5). Control variables: dynamic postarrival mechanisms (labor market status, legal status, language proficiency, and accommodation), gender, age, socioeconomic status before migration, arrival year, highest professional educational attainment, survey year fixed effects, federal state fixed effects, indicator for third person present at the interview, indicator for no one else except the interviewer present at the interview, dummies indicating the number of interviews (panel conditioning), and indicator for answering sensitive questions

*Data:* IAB-BAMF-SOEP Survey of Refugees, waves 1 to 4*.*

**Table S1** Information about cases excluded from the original IAB-BAMF-SOEP Survey of Refugees sample

|  | Person observations left after restriction | Person-year observations left after restriction |
| --- | --- | --- |
| **Stages of sample selection** |  |  |
| Original sample | 8,321 | 18,342 |
| Keep if |  |  |
| SYR, ERI, AFG, IRQ | 6831 | 15461 |
| with refugee questionnaire | 6772 | 15383 |
| aged 18-55 at the first interview | 6499 | 14819 |
| arrived between 2014-2016 | 5954 | 13684 |
| first interview maximum three years after arrival in Germany | 5758 | 13475 |
| first interview in 2016-2017 | 5399 | 12960 |
| participated at least in two waves | 3974 | 11535 |
| non-missing on dependent variable in the first interview | 3957 | 11464 |

*Data*: IAB-BAMF-SOEP Survey of Refugees, waves 1 to 4.

**Table S2** Descriptive statistics of outcome variables and the prearrival and socioeconomic variables

|  | Mean/ share | Std. Deviation | Min | Max |
| --- | --- | --- | --- | --- |
| **Prearrival mechanisms** |  |  |  |  |
| *Reasons for leaving* |  |  |  |  |
| None of these | 0.486 |  | 0 | 1 |
| Social reasons only | 0.062 |  | 0 | 1 |
| Economic reasons only | 0.313 |  | 0 | 1 |
| Economic and social reason | 0.131 |  | 0 | 1 |
| Missing information | 0.007 |  | 0 | 1 |
| *Number of different modes of transport^1,4^* | 3.928 | 1.689 | 0 | 9 |
| *Missing information on modes^4^* | 0.000 |  | 0 | 1 |
| *Financing of migration via family/friends^2,4^* | 0.447 |  | 0 | 1 |
| *Financing of migration marketing assets^2,4^* | 0.769 |  | 0 | 1 |
| *Missing information on financing^4^* | 0.014 |  | 0 | 1 |
| *Number of traumatic events^3,4^* | 1.078 | 1.326 | 0 | 7 |
| *Missing information events^4^* | 0.010 |  | 0 | 1 |
| *Responses to sensitive questions* | 0.625 |  | 0 | 1 |
| **Socioeconomic variables** |  |  |  |  |
| *Country of origin* |  |  |  |  |
| Afghanistan | 0.148 |  | 0 | 1 |
| Eritrea | 0.058 |  | 0 | 1 |
| Iraq | 0.147 |  | 0 | 1 |
| Syria | 0.646 |  | 0 | 1 |
| *Highest professional educational attainment* |  |  |  |  |
| No professional education | 0.768 |  | 0 | 1 |
| Vocational education | 0.048 |  | 0 | 1 |
| University | 0.170 |  | 0 | 1 |
| Other | 0.011 |  |  |  |
| Missing information | 0.005 |  | 0 | 1 |
| *Socioeconomic status before migration* |  |  |  |  |
| Low | 0.208 |  | 0 | 1 |
| Medium | 0.471 |  | 0 | 1 |
| High | 0.312 |  | 0 | 1 |
| Missing information | 0.009 |  | 0 | 1 |
| *Male gender* | 0.620 |  | 0 | 1 |
| *Age* | 32.273 | 9.500 | 18 | 55 |
| *Arrival Year* |  |  |  |  |
| 2014 | 0.175 |  | 0 | 1 |
| 2015 | 0.673 |  | 0 | 1 |
| 2016 | 0.151 |  | 0 | 1 |
| *Legal status* |  |  |  |  |
| In process | 0.236 |  | 0 | 1 |
| Protection granted | 0.687 |  | 0 | 1 |
| Protection denied | 0.021 |  | 0 | 1 |
| Other | 0.042 |  |  |  |
| *Labor market status* |  |  |  |  |
| Permanent work contract | 0.033 |  | 0 | 1 |
| Temporary work contract | 0.022 |  | 0 | 1 |
| Participating in education | 0.068 |  | 0 | 1 |
| Job-seeking | 0.173 |  | 0 | 1 |
| Inactive | 0.704 |  | 0 | 1 |
| *German language proficiency* |  |  |  |  |
| None at all | 0.103 |  | 0 | 1 |
| Not very good | 0.378 |  | 0 | 1 |
| Average | 0.351 |  | 0 | 1 |
| Good | 0.149 |  | 0 | 1 |
| Very good | 0.018 |  | 0 | 1 |
| Missing information | 0.001 |  | 0 | 1 |
| *Location of partner* |  |  |  |  |
| No partner | 0.317 |  | 0 | 1 |
| In Germany | 0.591 |  | 0 | 1 |
| Abroad | 0.084 |  | 0 | 1 |
| Missing information | 0.008 |  | 0 | 1 |
| *Type of residence* |  |  |  |  |
| Shared accommodation/other | 0.328 |  | 0 | 1 |
| Private flat/house | 0.668 |  | 0 | 1 |
| Missing information | 0.005 |  | 0 | 1 |
| *Third person present at interview* |  |  |  |  |
| Partner | 0.251 |  | 0 | 1 |
| Other third person | 0.264 |  | 0 | 1 |
| Nobody | 0.436 |  | 0 | 1 |
| Partner and other third person | 0.049 |  | 0 | 1 |
| *Federal state* |  |  |  |  |
| Schleswig-Holstein | 0.051 |  | 0 | 1 |
| Hamburg | 0.023 |  | 0 | 1 |
| Lower Saxony | 0.101 |  | 0 | 1 |
| Bremen | 0.013 |  | 0 | 1 |
| North Rhine-Westphalia | 0.212 |  | 0 | 1 |
| Hesse | 0.099 |  | 0 | 1 |
| Rhineland-Palatinate | 0.041 |  | 0 | 1 |
| Baden-Wuerttemberg | 0.126 |  | 0 | 1 |
| Bavaria | 0.131 |  | 0 | 1 |
| Saarland | 0.024 |  | 0 | 1 |
| Berlin | 0.042 |  | 0 | 1 |
| Brandenburg | 0.033 |  | 0 | 1 |
| Mecklenburg-Hither Pomerania | 0.015 |  | 0 | 1 |
| Saxony | 0.033 |  | 0 | 1 |
| Saxony-Anhalt | 0.024 |  | 0 | 1 |
| Thuringia | 0.029 |  | 0 | 1 |
| Missing information | 0.005 |  | 0 | 1 |
| *Wave* |  |  |  |  |
| Wave 1 | 0.600 |  | 0 | 1 |
| Wave 2 | 0.400 |  | 0 | 1 |
| N | 3957 |  |  |  |

*Note*. 1) if non-missing information on mode; 2) if non-missing information on financing; 3) if non-missing information on events; 4) if responsed to sensitive questions.

*Data*: IAB-BAMF-SOEP Survey of Refugees, waves 1 to 4.

**Table S3** Descriptive statistics of time-varying postarrival variables by individual survey participation (number of waves)

|  | 1 | 2 | 3 | 4 | Total |
| --- | --- | --- | --- | --- | --- |
| *Legal status* |  |  |  |  |  |
| In process | 23.60 | 10.95 | 9.12 | 4.94 | 14.39 |
| Protection granted | 68.71 | 83.10 | 85.44 | 89.09 | 79.17 |
| Protection denied | 2.10 | 2.23 | 2.74 | 2.67 | 2.34 |
| Other status | 4.17 | 2.48 | 1.08 | 1.34 | 2.65 |
| Missing information | 1.42 | 1.24 | 1.62 | 1.95 | 1.45 |
| *Labor market status* |  |  |  |  |  |
| Permanent work contract | 3.31 | 9.76 | 14.72 | 17.08 | 9.27 |
| Temporary work contract | 2.17 | 7.98 | 12.32 | 14.40 | 7.50 |
| Participating in education | 6.82 | 10.34 | 10.31 | 9.98 | 9.09 |
| Job-seeking | 17.31 | 19.31 | 19.51 | 18.93 | 18.63 |
| Inactive | 70.38 | 52.61 | 43.14 | 39.61 | 55.50 |
| *German language proficiency* |  |  |  |  |  |
| None at all | 10.26 | 2.94 | 1.62 | 1.44 | 5.04 |
| Not very good | 37.83 | 22.86 | 16.92 | 12.14 | 25.78 |
| Average | 35.13 | 38.22 | 40.02 | 40.02 | 37.71 |
| Good | 14.91 | 29.30 | 31.48 | 37.96 | 25.56 |
| Very good | 1.77 | 6.59 | 9.97 | 8.44 | 5.84 |
| Missing information | 0.10 | 0.10 | 0.00 | 0.00 | 0.07 |
| *Type of residence* |  |  |  |  |  |
| Shared accommodation/other | 32.75 | 17.38 | 12.55 | 9.67 | 20.94 |
| Private flat/house | 66.79 | 82.26 | 86.91 | 90.33 | 78.65 |
| Missing information | 0.45 | 0.35 | 0.54 | 0.00 | 0.40 |
| N | 3957 | 3946 | 2589 | 972 | 11464 |

*Data*: IAB-BAMF-SOEP Survey of Refugees, waves 1 to 4.

**Table S4** Descriptive statistics of the controls by individual survey participation (number of waves)

|  | 1 | 2 | 3 | 4 | Total |
| --- | --- | --- | --- | --- | --- |
| *Third person present at interview* |  |  |  |  |  |
| Partner | 25.09 | 17.61 | 17.27 | 13.68 | 19.78 |
| Other third person | 26.38 | 18.04 | 12.63 | 8.44 | 18.89 |
| Nobody | 43.64 | 53.37 | 62.77 | 73.56 | 53.85 |
| Partner and other third person | 4.88 | 10.97 | 7.34 | 4.32 | 7.48 |
| *Federal State* |  |  |  |  |  |
| Schleswig-Holstein | 5.13 | 5.04 | 4.98 | 5.66 | 5.11 |
| Hamburg | 2.30 | 2.38 | 2.09 | 2.26 | 2.28 |
| Lower Saxony | 10.06 | 10.29 | 9.46 | 8.13 | 9.84 |
| Bremen | 1.29 | 1.29 | 1.62 | 1.23 | 1.36 |
| North Rhine-Westphalia | 21.15 | 21.92 | 21.86 | 19.03 | 21.40 |
| Hesse | 9.93 | 10.09 | 10.27 | 8.44 | 9.94 |
| Rhineland-Palatinate | 4.09 | 4.08 | 4.13 | 2.67 | 3.98 |
| Baden-Wuerttemberg | 12.56 | 12.44 | 12.67 | 16.67 | 12.89 |
| Bavaria | 13.07 | 12.80 | 12.21 | 12.45 | 12.73 |
| Saarland | 2.40 | 2.26 | 2.36 | 3.40 | 2.42 |
| Berlin | 4.22 | 4.21 | 4.02 | 3.91 | 4.14 |
| Brandenburg | 3.31 | 3.22 | 3.09 | 4.01 | 3.29 |
| Mecklenburg-Hither Pomerania | 1.47 | 1.32 | 1.31 | 0.82 | 1.33 |
| Saxony | 3.29 | 3.27 | 3.51 | 4.63 | 3.45 |
| Saxony-Anhalt | 2.43 | 2.33 | 2.97 | 4.22 | 2.67 |
| Thuringia | 2.86 | 2.71 | 2.90 | 2.47 | 2.78 |
| Missing information | 0.45 | 0.35 | 0.54 | 0.00 | 0.40 |
| *Survey year* |  |  |  |  |  |
| 2016 | 59.97 | - | - | - | 20.70 |
| 2017 | 40.03 | 49.32 | - | - | 30.79 |
| 2018 | - | 41.41 | 51.37 | - | 25.85 |
| 2019 | - | 9.28 | 48.63 | 100.00 | 22.65 |
| N | 3957 | 3946 | 2589 | 972 | 11464 |

*Data*: IAB-BAMF-SOEP Survey of Refugees, waves 1 to 4.

**Table S5** Associations between prearrival experiences and life satisfaction and self-rated health at the first interview: Omitted controls

| **Panel A: LIFE SATISFACTION** |  |  |  |  |  |
| --- | --- | --- | --- | --- | --- |
|  | Model 1.3 | Model 1.4 | Model 1.5 | Model 1.6 | Model 1.7 |
| *Country of origin (ref. Syria)* |  |  |  |  |  |
| Afghanistan | 0.387** | 0.423*** | 0.404*** | 0.429*** | 0.425*** |
| Eritrea | 0.606*** | 0.615*** | 0.617*** | 0.684*** | 0.712*** |
| Iraq | 0.225* | 0.240* | 0.241* | 0.238* | 0.217* |
| *Survey wave at first interview (ref. 2016)* |  |  |  |  |  |
| 2017 | -0.033 | -0.01 | -0.034 | -0.036 | -0.017 |
| *Age* | -0.059* | -0.065* | -0.064* | -0.063* | -0.059* |
| *Age* squared | 0.001+ | 0.001* | 0.001+ | 0.001+ | 0.001+ |
| *Legal status (ref. In process)* |  |  |  |  |  |
| Protection granted | 0.407*** | 0.394*** | 0.400*** | 0.373*** | 0.377*** |
| Protection denied | 0.077 | 0.053 | 0.068 | 0.112 | 0.115 |
| Other status | 0.285 | 0.266 | 0.291 | 0.257 | 0.234 |
| Missing information | -0.424 | -0.455 | -0.442 | -0.477 | -0.499 |
| *Arrival year* (ref. 2016) |  |  |  |  |  |
| 2014 | -0.269+ | -0.296* | -0.266+ | -0.233 | -0.262+ |
| 2015 | -0.116 | -0.103 | -0.115 | -0.092 | -0.083 |
| *Third person present at interview* (ref. Partner) |  |  |  |  |  |
| Other third person | -0.349** | -0.359** | -0.362** | -0.364** | -0.354** |
| Nobody | -0.258* | -0.268** | -0.264** | -0.262** | -0.256* |
| Partner and other third person | -0.351+ | -0.339+ | -0.361+ | -0.358+ | -0.351+ |
| *Gender (ref. Female)* |  |  |  |  |  |
| Male | -0.09 | -0.089 | -0.094 | -0.085 | -0.067 |
| *Type of residence (ref. Shared accommodation)* |  |  |  |  |  |
| Private flat/house | 0.726*** | 0.722*** | 0.726*** | 0.723*** | 0.715*** |
| Missing information | 0.000 | 0.000 | 0.000 | 0.000 | 0.000 |
| *Highest professional educational attainment (ref. No professional education)* |  |  |  |  |  |
| Vocational education | -0.245 | -0.237 | -0.234 | -0.228 | -0.238 |
| University | -0.558*** | -0.545*** | -0.551*** | -0.549*** | -0.545*** |
| Other | 0.040 | 0.074 | 0.041 | 0.029 | 0.023 |
| Missing information | -0.448 | -0.552 | -0.420 | -0.408 | -0.548 |
| *German language proficiency (ref. None at all)* |  |  |  |  |  |
| Not very good | 0.105 | 0.108 | 0.098 | 0.118 | 0.127 |
| Average | 0.445*** | 0.444*** | 0.432** | 0.461*** | 0.474*** |
| Good | 0.399* | 0.390* | 0.382* | 0.409** | 0.416** |
| Very good | 0.915** | 0.934** | 0.922** | 0.935** | 0.907** |
| Missing information | 0.711 | 0.679 | 0.711 | 0.63 | 0.661 |
| *Socioeconomic status before migration (ref. Low)* |  |  |  |  |  |
| Medium | 0.148 | 0.164+ | 0.157+ | 0.147 | 0.149 |
| High | -0.047 | -0.018 | -0.03 | -0.021 | -0.029 |
| Missing information | 0.625 | 0.716+ | 0.669+ | 0.665+ | 0.643+ |
| *Residence of partner (ref. No partner)* |  |  |  |  |  |
| In Germany | 0.435*** | 0.428*** | 0.434*** | 0.429*** | 0.427*** |
| Abroad | -0.266+ | -0.267+ | -0.277+ | -0.258+ | -0.247 |
| Missing information | 0.269 | 0.287 | 0.307 | 0.258 | 0.212 |
| *Dummy for answering sensitive questions* | 0.435*** | 0.428*** | 0.434*** | 0.429*** | 0.427*** |
| Constant | 7.805*** | 7.515*** | 7.545*** | 7.534*** | 7.813*** |
| Number of persons | 3957 | 3957 | 3957 | 3957 | 3957 |
| Adj. R^2^ | 0.069 | 0.07 | 0.068 | 0.073 | 0.074 |
| F Statistic | 6.546 | 6.823 | 6.551 | 7.097 | 6.252 |
|  |  |  |  |  |  |
| **Panel B: SELF-RATED HEALTH** |  |  |  |  |  |
|  | Model 2.3 | Model 2.4 | Model 2.5 | Model 2.6 | Model 2.7 |
| *Country of origin (ref. Syria)* |  |  |  |  |  |
| Afghanistan | -0.132* | -0.119* | -0.124* | -0.107* | -0.115* |
| Eritrea | 0.227** | 0.225** | 0.232** | 0.277*** | 0.295*** |
| Iraq | -0.087+ | -0.075 | -0.079 | -0.08 | -0.093+ |
| *Survey wave at first interview (ref. 2016)* |  |  |  |  |  |
| 2017 | -0.058 | -0.053 | -0.059 | -0.060 | -0.060 |
| *Age* | 0.002 | 0.000 | -0.001 | 0.001 | 0.002 |
| *Age squared* | -0.000* | -0.000* | -0.000* | -0.000* | -0.000** |
| *Legal status (ref. In process)* |  |  |  |  |  |
| Protection granted | 0.093* | 0.091* | 0.091* | 0.074+ | 0.074+ |
| Protection denied | 0.008 | 0.004 | 0.003 | 0.037 | 0.037 |
| Other | 0.001 | -0.002 | 0.002 | -0.021 | -0.022 |
| Missing information | -0.064 | -0.065 | -0.062 | -0.095 | -0.093 |
| *Arrival year* (ref. 2016) |  |  |  |  |  |
| 2014 | -0.097 | -0.099 | -0.09 | -0.074 | -0.070 |
| 2015 | 0.029 | 0.030 | 0.030 | 0.044 | 0.048 |
| *Third person present at interview* (ref. Partner) |  |  |  |  |  |
| Other third person | -0.117* | -0.121* | -0.122* | -0.123* | -0.123* |
| Nobody | -0.158*** | -0.160*** | -0.159*** | -0.158*** | -0.155*** |
| Partner and other third person | -0.115 | -0.111 | -0.118 | -0.117 | -0.121 |
| *Gender (ref. Female)* |  |  |  |  |  |
| Male | 0.293*** | 0.293*** | 0.291*** | 0.300*** | 0.302*** |
| *Type of residence* (ref. Shared accommodation) |  |  |  |  |  |
| Private flat/house | 0.055 | 0.054 | 0.055 | 0.051 | 0.051 |
| Missing information | 0.000 | 0.000 | 0.000 | 0.000 | 0.000 |
| *Highest professional educational attainment (ref. No professional education)* |  |  |  |  |  |
| Vocational education | 0.023 | 0.028 | 0.026 | 0.033 | 0.025 |
| University | 0.036 | 0.04 | 0.037 | 0.042 | 0.037 |
| Other | 0.165 | 0.175 | 0.166 | 0.156 | 0.145 |
| Missing information | -0.844*** | -0.907*** | -0.836*** | -0.824*** | -0.898*** |
| *German language proficiency (ref. None at all)* |  |  |  |  |  |
| Not very good | 0.101+ | 0.100+ | 0.096+ | 0.112* | 0.108+ |
| Average | 0.231*** | 0.228*** | 0.223*** | 0.246*** | 0.244*** |
| Good | 0.376*** | 0.369*** | 0.365*** | 0.385*** | 0.385*** |
| Very good | 0.562*** | 0.566*** | 0.572*** | 0.569*** | 0.566*** |
| Missing information | 0.151 | 0.158 | 0.175 | 0.114 | 0.127 |
| *Socioeconomic status before migration (ref. Low)* |  |  |  |  |  |
| Medium | 0.104* | 0.112** | 0.107* | 0.103* | 0.098* |
| High | 0.112* | 0.125** | 0.118* | 0.127** | 0.117* |
| Missing information | 0.345* | 0.367* | 0.359* | 0.347* | 0.351* |
| *Residence of partner (ref. No partner)* |  |  |  |  |  |
| In Germany | 0.037 | 0.036 | 0.034 | 0.035 | 0.031 |
| Abroad | -0.028 | -0.031 | -0.033 | -0.021 | -0.016 |
| Missing information | -0.193 | -0.181 | -0.183 | -0.209 | -0.23 |
| *Dummy for answering sensitive questions* | 0.054 | 0.114+ | 0.103 | 0.169*** | 0.148+ |
| Constant | 4.077*** | 3.994*** | 4.018*** | 3.999*** | 4.096*** |
| Number of persons | 3957 | 3957 | 3957 | 3957 | 3957 |
| Adj. R^2^ | 0.131 | 0.131 | 0.131 | 0.14 | 0.141 |
| F Statistic | 12.281 | 12.681 | 12.485 | 13.654 | 11.798 |

*Note*. Statistical significance at: + p < 0.10, * p < 0.05, ** p < 0.01, *** p < 0.001. OLS regression coefficients. Dependent variables: life satisfaction (0 to 10) and self-rated health (1 to 5). Further controls not shown: federal state fixed effects.

*Data*: IAB-BAMF-SOEP Survey of Refugees, waves 1 to 4.

**Table S6** Associations between postarrival experiences and life satisfaction and self-rated health: Omitted controls

| **Panel A: LIFE SATISFACTION** | | | | | |
| --- | --- | --- | --- | --- | --- |
|  | Model 3.1 | Model 3.2 | Model 3.3 | Model 3.4 | Model 3.5 |
| *Wave dummy (ref. wave 1)* |  |  |  |  |  |
| Wave 2 | 0.336** | 0.336** | 0.316** | 0.338** | 0.275* |
| Wave 3 | 0.486* | 0.466* | 0.440* | 0.465* | 0.370+ |
| Wave 4 | 0.588* | 0.553* | 0.512+ | 0.552* | 0.411 |
| *Age* | 0.015 | 0.006 | 0.004 | -0.001 | -0.024 |
| *Age squared* | 0.000 | 0.000 | 0.000 | 0.000 | 0.001 |
| *Federal state (ref. Schleswig-Holstein)* |  |  |  |  |  |
| Hamburg | 0.558 | 0.523 | 0.584 | 0.704 | 0.657 |
| Lower Saxony | 0.235 | 0.222 | 0.204 | 0.256 | 0.21 |
| Bremen | 0.538 | 0.598 | 0.448 | 0.424 | 0.373 |
| North Rhine-Westphalia | -0.455 | -0.458 | -0.469 | -0.403 | -0.417 |
| Hesse | -0.541 | -0.532 | -0.534 | -0.457 | -0.44 |
| Rhineland-Palatinate | -2.104 | -2.083 | -2.068 | -2.092 | -2.037 |
| Baden-Wuerttemberg | -0.541 | -0.569 | -0.567 | -0.409 | -0.387 |
| Bavaria | 0.593 | 0.558 | 0.552 | 0.71 | 0.663 |
| Saarland | -1.135+ | -1.107+ | -1.117+ | -1.182* | -1.162* |
| Berlin | 0.028 | -0.064 | 0.005 | 0.186 | 0.149 |
| Brandenburg | 1.538* | 1.540* | 1.553* | 1.676* | 1.597* |
| Mecklenburg-Hither Pomerania | 0.409 | 0.373 | 0.33 | 0.436 | 0.496 |
| Saxony | 0.701 | 0.653 | 0.595 | 0.687 | 0.560 |
| Saxony-Anhalt | 0.619 | 0.625 | 0.598 | 0.606 | 0.608 |
| Thuringia | -0.593 | -0.63 | -0.649 | -0.63 | -0.681 |
| Missing information | -0.392 | -0.404 | -0.427 | 0.083 | 0.056 |
| *Third person present at interview* (ref. Partner) |  |  |  |  |  |
| Other third person | -0.155* | -0.152* | -0.151* | -0.155* | -0.148* |
| Nobody | -0.255*** | -0.252*** | -0.249*** | -0.247*** | -0.243*** |
| Partner and other third person | -0.184* | -0.188* | -0.192* | -0.188* | -0.185* |
| *Panel Conditioning (ref. first interview)* |  |  |  |  |  |
| 2nd interview | -0.367** | -0.361** | -0.393*** | -0.414*** | -0.443*** |
| 3rd interview | -0.544** | -0.540** | -0.573** | -0.600*** | -0.625*** |
| 4th interview | -0.652** | -0.645** | -0.675** | -0.708** | -0.719** |
| Constant | 7.029*** | 7.202*** | 7.069*** | 6.918*** | 7.110*** |
| Person-years | 11464 | 11464 | 11464 | 11464 | 11464 |
| Number of persons | 3957 | 3957 | 3957 | 3957 | 3957 |
| R^2^ overall | 0.002 | 0.002 | 0.002 | 0.006 | 0.009 |
| R^2^ within | 0.009 | 0.01 | 0.01 | 0.014 | 0.018 |
|  |  |  |  |  |  |
| **Panel B: SELF-RATED HEALTH** | | | | | |
|  | Model 4.1 | Model 4.2 | Model 4.3 | Model 4.4 | Model 4.5 |
| *Wave dummy (ref. wave 1)* |  |  |  |  |  |
| Wave 2 | 0.086 | 0.088+ | 0.081 | 0.096+ | 0.061 |
| Wave 3 | 0.186* | 0.179* | 0.174+ | 0.197* | 0.141 |
| Wave 4 | 0.142 | 0.129 | 0.117 | 0.152 | 0.077 |
| *Age* | 0.071* | 0.068* | 0.067* | 0.072* | 0.057+ |
| *Age squared* | -0.001** | -0.001** | -0.001* | -0.001** | -0.001* |
| *Federal state (ref. Schleswig-Holstein)* |  |  |  |  |  |
| Hamburg | 0.430+ | 0.418+ | 0.434+ | 0.439+ | 0.437+ |
| Lower Saxony | 0.179 | 0.186 | 0.168 | 0.182 | 0.174 |
| Bremen | 0.034 | 0.044 | 0.003 | 0.036 | -0.018 |
| North Rhine-Westphalia | 0.029 | 0.036 | 0.026 | 0.035 | 0.033 |
| Hesse | 0.451+ | 0.460+ | 0.458+ | 0.462+ | 0.466+ |
| Rhineland-Palatinate | -0.452 | -0.424 | -0.432 | -0.448 | -0.412 |
| Baden-Wuerttemberg | -0.03 | -0.034 | -0.035 | -0.023 | -0.014 |
| Bavaria | 0.033 | 0.036 | 0.025 | 0.049 | 0.027 |
| Saarland | -0.177 | -0.168 | -0.159 | -0.169 | -0.179 |
| Berlin | 0.249 | 0.232 | 0.258 | 0.27 | 0.244 |
| Brandenburg | 0.38 | 0.371 | 0.379 | 0.394 | 0.368 |
| Mecklenburg-Hither Pomerania | 0.200 | 0.230 | 0.199 | 0.213 | 0.223 |
| Saxony | -0.706 | -0.706 | -0.743 | -0.701 | -0.754 |
| Saxony-Anhalt | 0.033 | 0.047 | 0.034 | 0.037 | 0.036 |
| Thuringia | -0.133 | -0.148 | -0.159 | -0.142 | -0.161 |
| Missing information | -0.053 | -0.054 | -0.06 | -0.008 | -0.03 |
| *Third person present at interview* (ref. Partner) |  |  |  |  |  |
| Other third person | -0.014 | -0.014 | -0.012 | -0.014 | -0.012 |
| Nobody | -0.036 | -0.034 | -0.031 | -0.033 | -0.034 |
| Partner and other third person | 0.065 | 0.062 | 0.061 | 0.062 | 0.064 |
| *Panel Conditioning (ref. first interview)* |  |  |  |  |  |
| 2nd interview | -0.092+ | -0.094+ | -0.104* | -0.096+ | -0.110* |
| 3rd interview | -0.149+ | -0.152+ | -0.162+ | -0.154+ | -0.168+ |
| 4th interview | -0.164 | -0.164 | -0.174 | -0.168 | -0.178 |
| Constant | 2.898*** | 2.950*** | 2.917*** | 2.862*** | 3.010*** |
| Person-years | 11464 | 11464 | 11464 | 11464 | 11464 |
| Number of persons | 3957 | 3957 | 3957 | 3957 | 3957 |
| R^2^ overall | 0.029 | 0.03 | 0.032 | 0.023 | 0.044 |
| R^2^ within | 0.009 | 0.009 | 0.01 | 0.008 | 0.012 |

*Note*. Statistical significance at: + p < 0.10, * p < 0.05, ** p < 0.01, *** p < 0.001. FE regression coefficients. Dependent variables: Overall life satisfaction (0 to 10) and self-rated health (1 to 5).

*Data*: IAB-BAMF-SOEP Survey of Refugees, waves 1 to 4.

**Table S7** Life satisfaction and postarrival experiences over time

| **LIFE SATISFACTION** |  |  |  |  |  |
| --- | --- | --- | --- | --- | --- |
|  | Model S7.1 | Model S7.2 | Model S7.3 | Model S7.4 | Model S7.5 |
| *Labor market status (ref. Never received a permanent work contract)* |  |  |  |  |  |
| Permanent work contract at first interview | 0.214+ | 0.257 |  |  |  |
| Transition to permanent work contract | 0.149* | 0.125 |  |  |  |
| *Legal status (ref. Never granted* protection *status)* |  |  |  |  |  |
| Granted protection at first interview | 0.839*** |  | 0.833*** |  |  |
| Transition to granted protection | 0.673*** |  | 0.479** |  |  |
| *Language proficiency* (ref. Never good proficiency) |  |  |  |  |  |
| Good proficiency at first interview | 0.181* |  |  | 0.283** |  |
| Transition to good proficiency | 0.118* |  |  | 0.141+ |  |
| *Accommodation* (ref. Never in private flat) |  |  |  |  |  |
| In private flat at first interview | 0.947*** |  |  |  | 1.105*** |
| Transition to private flat | 0.622*** |  |  |  | 0.363* |
| *Years since first interview* (ref. 0) |  |  |  |  |  |
| 1 year since first interview | -0.122 | -0.166* | -0.441* | -0.133 | -0.370* |
| 2 years since first interview | -0.05 | -0.095 | -0.368 | -0.063 | -0.340 |
| 3 years since first interview | 0.053 | -0.053 | -0.164 | -0.082 | -0.199 |
| *Interactions of mechanisms and time dummies* |  |  |  |  |  |
| 1 year # Permanent work contract at first interview |  | -0.149 |  |  |  |
| 1 year # Transition to permanent work contract |  | 0.089 |  |  |  |
| 2 years # Permanent work contract at first interview |  | -0.104 |  |  |  |
| 2 years # Transition to permanent work contract |  | 0.005 |  |  |  |
| 3 years # Permanent work contract at first interview |  | 0.144 |  |  |  |
| 3 years # Transition to permanent work contract |  | 0.128 |  |  |  |
| 1 year # Granted protection at first interview |  |  | 0.394* |  |  |
| 1 year # Transition to granted protection |  |  | 0.391* |  |  |
| 2 years # Granted protection at first interview |  |  | 0.359+ |  |  |
| 2 years # Transition to granted protection |  |  | 0.601** |  |  |
| 3 years # Granted protection at first interview |  |  | 0.207 |  |  |
| 3 years # Transition to granted protection |  |  | 0.558* |  |  |
| 1 year # Good proficiency at first interview |  |  |  | -0.125 |  |
| 1 year # Transition to good proficiency |  |  |  | 0.019 |  |
| 2 years # Good proficiency at first interview |  |  |  | 0.023 |  |
| 2 years # Transition to good proficiency |  |  |  | -0.064 |  |
| 3 years # Good proficiency at first interview |  |  |  | 0.227 |  |
| 3 years # Transition to good proficiency |  |  |  | 0.141 |  |
| 1 year # In private flat at first interview |  |  |  |  | 0.096 |
| 1 year # Transition to private flat |  |  |  |  | 0.680*** |
| 2 years # In private flat at first interview |  |  |  |  | 0.096 |
| 2 years # Transition to private flat |  |  |  |  | 0.666*** |
| 3 years # In private flat at first interview |  |  |  |  | -0.032 |
| 3 years # Transition to private flat |  |  |  |  | 0.714** |
| **Controls** |  |  |  |  |  |
| *Country of origin* (ref. Syria) |  |  |  |  |  |
| Afghanistan | 0.427*** | -0.025 | 0.295*** | -0.009 | 0.206** |
| Eritrea | 0.412*** | 0.160 | 0.270** | 0.202+ | 0.413*** |
| Iraq | 0.244** | -0.000 | 0.209** | 0.005 | 0.071 |
| *Wave dummy* (ref. wave 1) |  |  |  |  |  |
| Wave 2 | 0.142+ | 0.175* | 0.105 | 0.161* | 0.170* |
| Wave 3 | 0.070 | 0.129 | -0.003 | 0.109 | 0.142 |
| Wave 4 | 0.003 | 0.092 | -0.057 | 0.052 | 0.112 |
| *Age* | 0.011 | 0.019 | 0.016 | 0.028 | 0.004 |
| *Age* squared | -0.000 | -0.000 | -0.000 | -0.000 | -0.000 |
| *Federal state* (ref. Schleswig-Holstein) |  |  |  |  |  |
| Hamburg | 0.129 | -0.078 | -0.136 | -0.046 | 0.230 |
| Lower Saxony | 0.026 | 0.116 | 0.092 | 0.112 | 0.063 |
| Bremen | -0.328+ | -0.109 | -0.273 | -0.062 | -0.241 |
| North Rhine-Westphalia | -0.005 | 0.050 | -0.015 | 0.079 | 0.050 |
| Hesse | -0.064 | -0.128 | -0.162 | -0.082 | -0.006 |
| Rhineland-Palatinate | -0.462** | -0.407* | -0.472** | -0.370* | -0.416* |
| Baden-Wuerttemberg | 0.117 | 0.040 | 0.022 | 0.064 | 0.171 |
| Bavaria | 0.181 | 0.148 | 0.086 | 0.171 | 0.271* |
| Saarland | -0.056 | 0.103 | 0.016 | 0.133 | 0.053 |
| Berlin | -0.249 | -0.462** | -0.542** | -0.433* | -0.158 |
| Brandenburg | -0.179 | -0.202 | -0.272 | -0.186 | -0.154 |
| Mecklenburg-Hither Pomerania | -0.054 | 0.100 | -0.001 | 0.113 | -0.003 |
| Saxony | 0.044 | 0.146 | 0.034 | 0.161 | 0.129 |
| Saxony-Anhalt | 0.248 | 0.448** | 0.329* | 0.426** | 0.319* |
| Thuringia | -0.459** | -0.323+ | -0.410* | -0.313+ | -0.404* |
| Missing information | -0.191 | -0.037 | -0.109 | -0.002 | 0.075 |
| *Socioeconomic status before migration* (ref. Low) |  |  |  |  |  |
| Medium | 0.084 | 0.137* | 0.111+ | 0.134* | 0.107 |
| High | -0.056 | 0.023 | -0.023 | 0.007 | -0.009 |
| Missing information | 0.002 | -0.018 | 0.036 | -0.012 | 0.013 |
| *Arrival year* (ref. 2016) |  |  |  |  |  |
| 2014 | 0.093 | 0.318*** | 0.275** | 0.274** | 0.154 |
| 2015 | 0.016 | 0.098 | 0.075 | 0.079 | 0.031 |
| *Third person present at interview* (ref. Partner) |  |  |  |  |  |
| Other third person | -0.289*** | -0.328*** | -0.316*** | -0.334*** | -0.286*** |
| Nobody | -0.359*** | -0.387*** | -0.380*** | -0.393*** | -0.337*** |
| Partner and other third person | -0.132 | -0.168* | -0.151+ | -0.179* | -0.146+ |
| *Gender* (ref. Female) |  |  |  |  |  |
| Male | -0.162** | -0.224*** | -0.166** | -0.217*** | -0.106* |
| *Highest professional educational attainment* (ref. No professional education) |  |  |  |  |  |
| Vocational education | -0.082 | -0.029 | -0.058 | -0.043 | -0.027 |
| University | -0.431*** | -0.348*** | -0.368*** | -0.401*** | -0.365*** |
| Other | 0.141 | 0.105 | 0.160 | 0.121 | 0.166 |
| Missing information | -0.007 | -0.169 | -0.161 | -0.173 | 0.018 |
| *Dummy for answering sensitive questions* | 0.051 | 0.065 | 0.060 | 0.061 | 0.073 |
| *Ever missing legal status* | 0.046 |  | 0.103 |  |  |
| *Ever missing language proficiency* | 0.165 |  |  | 0.044 |  |
| *Ever missing accommodation* | 0.271 |  |  |  | 0.262 |
| Constant | 5.787*** | 7.218*** | 6.636*** | 6.961*** | 6.587*** |
| Person-years | 11464 | 11464 | 11464 | 11464 | 11464 |
| Number of persons | 3957 | 3957 | 3957 | 3957 | 3957 |
| R^2^ overall | 0.059 | 0.028 | 0.045 | 0.029 | 0.051 |
| R^2^ within | 0.004 | 0.005 | 0.007 | 0.005 | 0.012 |

*Note*. Statistical significance at: + p < 0.10, * p < 0.05, ** p < 0.01, *** p < 0.001. REGC regression coefficients. Figures 1 and 2 are based on these regressions. Dependent variables: overall life satisfaction (0 to 10).

*Data*: IAB-BAMF-SOEP Survey of Refugees, waves 1 to 4.

**Table S8** Self-rated health and postarrival experiences over time

| **SELF-RATED HEALTH** |  |  |  |  |  |
| --- | --- | --- | --- | --- | --- |
|  | Model S8.1 | Model S8.2 | Model S8.3 | Model S8.4 | Model S8.5 |
| *Labor market status* (ref. Never received permanent work contract) |  |  |  |  |  |
| Permanent work contract at first interview | 0.054 | 0.128 |  |  |  |
| Transition to permanent work contract | 0.174*** | 0.175*** |  |  |  |
| *Legal status* (ref. Never granted protection status) |  |  |  |  |  |
| Granted protection at first interview | 0.045 |  | 0.116+ |  |  |
| Transition to granted protection | -0.016 |  | -0.025 |  |  |
| *Language proficiency* (ref. Never good proficiency) |  |  |  |  |  |
| Good proficiency at first interview | 0.247*** |  |  | 0.313*** |  |
| Transition to good proficiency | 0.188*** |  |  | 0.161*** |  |
| *Accommodation* (ref. Never in private flat) |  |  |  |  |  |
| In private flat at first interview | 0.175*** |  |  |  | 0.238*** |
| Transition to private flat | 0.128* |  |  |  | 0.154* |
| *Years since first interview* (ref. 0) |  |  |  |  |  |
| 1 year since first interview | 0.058 | 0.049 | 0.088 | 0.048 | 0.083 |
| 2 years since first interview | 0.155* | 0.128* | 0.220* | 0.124+ | 0.152 |
| 3 years since first interview | 0.266** | 0.217* | 0.313* | 0.205* | 0.138 |
| *Interactions of mechanisms and time dummies* |  |  |  |  |  |
| 1 year # Permanent work contract at first interview |  | -0.097 |  |  |  |
| 1 year # Transition to permanent work contract |  | -0.001 |  |  |  |
| 2 years # Permanent work contract at first interview |  | -0.111 |  |  |  |
| 2 years # Transition to permanent work contract |  | 0.044 |  |  |  |
| 3 years # Permanent work contract at first interview |  | -0.030 |  |  |  |
| 3 years # Transition to permanent work contract |  | 0.063 |  |  |  |
| 1 year # Granted protection at first interview |  |  | -0.034 |  |  |
| 1 year # Transition to granted protection |  |  | 0.035 |  |  |
| 2 years # Granted protection at first interview |  |  | -0.065 |  |  |
| 2 years # Transition to granted protection |  |  | 0.029 |  |  |
| 3 years # Granted protection at first interview |  |  | -0.050 |  |  |
| 3 years # Transition to granted protection |  |  | 0.079 |  |  |
| 1 year # Good proficiency at first interview |  |  |  | -0.104* |  |
| 1 year # Transition to good proficiency |  |  |  | 0.065 |  |
| 2 years # Good proficiency at first interview |  |  |  | -0.049 |  |
| 2 years # Transition to good proficiency |  |  |  | 0.066 |  |
| 3 years # Good proficiency at first interview |  |  |  | -0.006 |  |
| 3 years # Transition to good proficiency |  |  |  | 0.096+ |  |
| 1 year # In private flat at first interview |  |  |  |  | -0.054 |
| 1 year # Transition to private flat |  |  |  |  | -0.006 |
| 2 years # In private flat at first interview |  |  |  |  | -0.040 |
| 2 years # Transition to private flat |  |  |  |  | 0.031 |
| 3 years # In private flat at first interview |  |  |  |  | 0.093 |
| 3 years # Transition to private flat |  |  |  |  | 0.125 |
| **Controls** |  |  |  |  |  |
| *Country of origin* (ref. Syria) |  |  |  |  |  |
| Afghanistan | -0.059 | -0.136*** | -0.097* | -0.124*** | -0.096* |
| Eritrea | 0.227*** | 0.172*** | 0.223*** | 0.212*** | 0.250*** |
| Iraq | -0.081* | -0.118** | -0.098** | -0.112** | -0.108** |
| *Wave dummy* (ref. wave 1) |  |  |  |  |  |
| Wave 2 | -0.018 | -0.000 | -0.032 | -0.017 | -0.000 |
| Wave 3 | -0.040 | -0.008 | -0.059 | -0.025 | -0.007 |
| Wave 4 | -0.200* | -0.150+ | -0.220* | -0.175* | -0.151+ |
| *Age* | 0.017+ | 0.009 | 0.010 | 0.021* | 0.008 |
| *Age* *squared* | -0.001*** | -0.001*** | -0.001*** | -0.001*** | -0.001*** |
| *Federal state* (ref. Schleswig-Holstein) |  |  |  |  |  |
| Hamburg | 0.308*** | 0.247** | 0.238** | 0.280*** | 0.305*** |
| Lower Saxony | 0.200** | 0.223*** | 0.226*** | 0.211** | 0.215** |
| Bremen | 0.173 | 0.164 | 0.144 | 0.211+ | 0.141 |
| North Rhine-Westphalia | 0.030 | 0.014 | 0.012 | 0.044 | 0.016 |
| Hesse | 0.204** | 0.164* | 0.174** | 0.213** | 0.194** |
| Rhineland-Palatinate | -0.059 | -0.080 | -0.089 | -0.048 | -0.081 |
| Baden-Wuerttemberg | -0.007 | -0.034 | -0.022 | -0.014 | -0.003 |
| Bavaria | 0.082 | 0.062 | 0.069 | 0.082 | 0.093 |
| Saarland | -0.097 | -0.075 | -0.073 | -0.058 | -0.070 |
| Berlin | -0.080 | -0.154+ | -0.154+ | -0.122 | -0.098 |
| Brandenburg | 0.135 | 0.112 | 0.092 | 0.127 | 0.116 |
| Mecklenburg-Hither Pomerania | 0.090 | 0.100 | 0.078 | 0.107 | 0.070 |
| Saxony | -0.065 | -0.077 | -0.090 | -0.061 | -0.082 |
| Saxony-Anhalt | 0.014 | 0.056 | 0.024 | 0.033 | 0.012 |
| Thuringia | -0.142+ | -0.123 | -0.129 | -0.118 | -0.132 |
| Missing information | -0.124 | -0.079 | -0.080 | -0.062 | -0.112 |
| *Socioeconomic status before migration* (ref. Low) |  |  |  |  |  |
| Medium | 0.109*** | 0.119*** | 0.118*** | 0.115*** | 0.114*** |
| High | 0.098** | 0.123*** | 0.117** | 0.104** | 0.116** |
| Missing information | 0.150 | 0.164 | 0.167 | 0.155 | 0.173 |
| *Arrival year* (ref. 2016) |  |  |  |  |  |
| 2014 | -0.054 | 0.033 | 0.021 | -0.015 | 0.010 |
| 2015 | -0.017 | 0.018 | 0.009 | -0.003 | 0.006 |
| *Third person present at interview* (ref. Partner) |  |  |  |  |  |
| Other third person | -0.060* | -0.059* | -0.060* | -0.064* | -0.051+ |
| Nobody | -0.067** | -0.064** | -0.061* | -0.072** | -0.052* |
| Partner and other third person | 0.031 | 0.029 | 0.028 | 0.021 | 0.032 |
| *Gender* (ref. female) |  |  |  |  |  |
| Male | 0.248*** | 0.266*** | 0.306*** | 0.268*** | 0.321*** |
| *Highest professional educational attainment* (ref. No professional education) |  |  |  |  |  |
| Vocational education | 0.021 | 0.045 | 0.051 | 0.029 | 0.052 |
| University | 0.037 | 0.103** | 0.107** | 0.043 | 0.105** |
| Other | 0.139 | 0.159+ | 0.192* | 0.166 | 0.194+ |
| Missing information | -0.385+ | -0.411+ | -0.398+ | -0.409+ | -0.359 |
| *Dummy for answering sensitive questions* | 0.020 | 0.031 | 0.036 | 0.026 | 0.037 |
| *Ever missing legal status* | -0.054 |  | -0.036 |  |  |
| *Ever missing language proficiency* | -0.087 |  |  | -0.113 |  |
| *Ever missing accommodation* | 0.123 |  |  |  | 0.147 |
| Constant | 3.678*** | 4.063*** | 4.003*** | 3.782*** | 3.879*** |
| Person-years | 11464 | 11464 | 11464 | 11464 | 11464 |
| Number of persons | 3957 | 3957 | 3957 | 3957 | 3957 |
| R^2^ overall | 0.148 | 0.136 | 0.133 | 0.142 | 0.135 |
| R^2^ within | 0.006 | 0.006 | 0.006 | 0.007 | 0.006 |

*Note*. Statistical significance at: + p < 0.10, * p < 0.05, ** p < 0.01, *** p < 0.001. REGC regression coefficients. Figures 1 and 3 are based on these regressions. Dependent variables: self-rated health (1 to 5).

*Data*: IAB-BAMF-SOEP Survey of Refugees, waves 1 to 4.
