# Supplementary material for: The Impact of Pre- and Postarrival Mechanisms on Self-rated Health and Life Satisfaction Among Refugees in Germany
Source: Front Sociol. 2021 Jul 6;6:693518. doi: 10.3389/fsoc.2021.693518 (PMC8289889; doi:10.3389/fsoc.2021.693518)
Supplement: Supplementary file 2 [file DataSheet1.PDF]

Supplementary Material

Figure S1 Development of life satisfaction over time

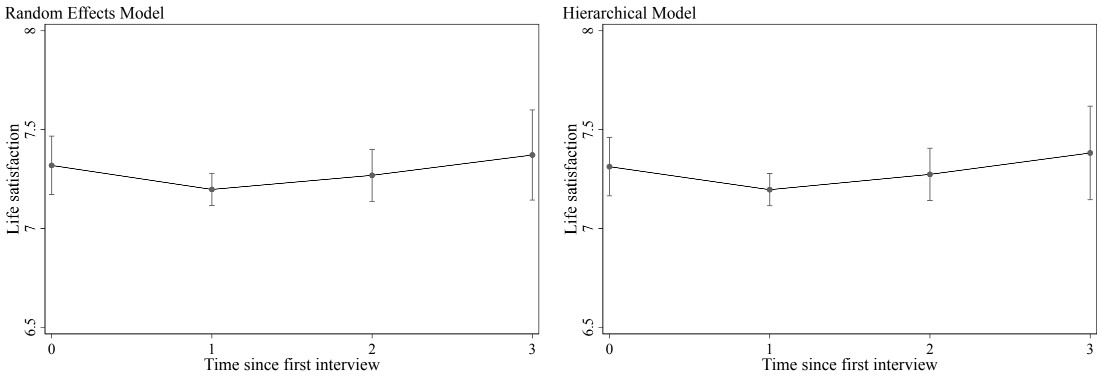

Note. Conditional profile plots estimated by a linear random effects growth curve model of life satisfaction as presented in the manuscript (left-hand side) and hierarchical random effects growth curve model of life satisfaction (right-hand side). Dependent variables: Overall life satisfaction (0 to 10). Control variables: dynamic postarrival mechanisms (labor market status, legal status, language proficiency, and accommodation), gender, age, socioeconomic status before migration, arrival year, highest professional educational attainment, survey year fixed effects, federal state fixed effects, indicator for third person present at the interview, indicator for no one else except the interviewer present at the interview, dummies indicating the number of interviews (panel conditioning), and indicator for answering sensitive questions.

Data: IAB-BAMF-SOEP Survey of Refugees, waves 1 to 4.

Figure S2: Development of self-rated health over time

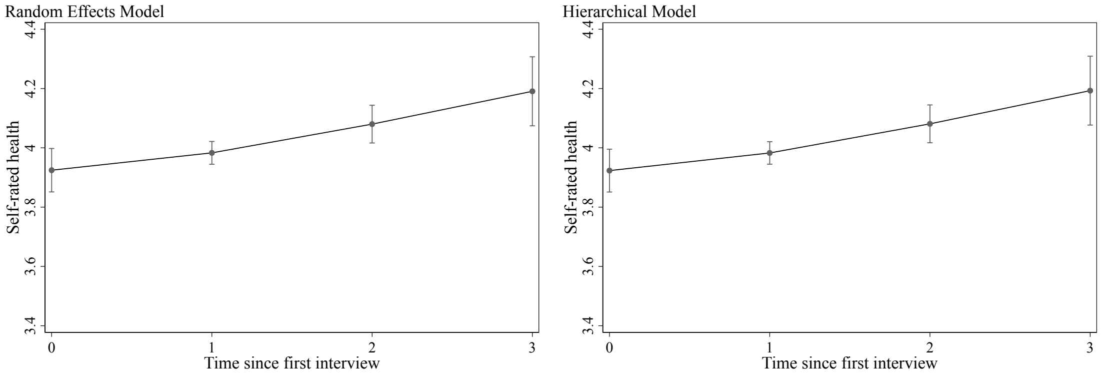

Note. Conditional profile plots estimated by a linear random effects growth curve model of self-rated health as presented in the manuscript (left-hand side) and hierarchical random effects growth curve model of self-rated health (right-hand side). Dependent variables: Self-rated health (1 to 5). Control variables: dynamic postarrival mechanisms (labor market status, legal status, language proficiency, and accommodation), gender, age, socioeconomic status before migration, arrival year, highest professional educational attainment, survey year fixed effects, federal state fixed effects, indicator for third person present at the interview, indicator for no one else except the interviewer present at the interview, dummies indicating the number of interviews (panel conditioning), and indicator for answering sensitive questions

Data: IAB-BAMF-SOEP Survey of Refugees, waves 1 to 4.

Table S1 Information about cases excluded from the original IAB-BAMF-SOEP Survey of Refugees sample

	Person observations left after restriction	Person-year observations left after restriction
**Stages of sample selection**		
Original sample	8,321	18,342
Keep if		
SYR, ERI, AFG, IRQ	6831	15461
with refugee questionnaire	6772	15383
aged 18-55 at the first interview	6499	14819
arrived between 2014-2016	5954	13684
first interview maximum three years after arrival in Germany	5758	13475
first interview in 2016-2017	5399	12960
participated at least in two waves	3974	11535
non-missing on dependent variable in the first interview	3957	11464

Data: IAB-BAMF-SOEP Survey of Refugees, waves 1 to 4.

Table S2 Descriptive statistics of outcome variables and the prearrival and socioeconomic variables

	Mean/ share	Std. Deviation	Min	Max
**Prearrival mechanisms**				
Reasons for leaving				
None of these	0.486		0	1
Social reasons only	0.062		0	1
Economic reasons only	0.313		0	1
Economic and social reason	0.131		0	1
Missing information	0.007		0	1
Number of different modes of transport[1,4]	3.928	1.689	0	9
Missing information on modes[4]	0.000		0	1
Financing of migration via family/friends[2,4]	0.447		0	1
Financing of migration marketing assets[2,4]	0.769		0	1
Missing information on financing[4]	0.014		0	1
Number of traumatic events[3,4]	1.078	1.326	0	7
Missing information events[4]	0.010		0	1
Responses to sensitive questions	0.625		0	1
**Socioeconomic variables**				
Country of origin				
Afghanistan	0.148		0	1
Eritrea	0.058		0	1
Iraq	0.147		0	1
Syria	0.646		0	1
Highest professional educational attainment				
No professional education	0.768		0	1
Vocational education	0.048		0	1
University	0.170		0	1
Other	0.011			
Missing information	0.005		0	1
Socioeconomic status before migration				
Low	0.208		0	1
Medium	0.471		0	1
High	0.312		0	1
Missing information	0.009		0	1
Male gender	0.620		0	1
Age	32.273	9.500	18	55
Arrival Year				
2014	0.175		0	1
2015	0.673		0	1
2016	0.151		0	1
Legal status				
In process	0.236		0	1
Protection granted	0.687		0	1
Protection denied	0.021		0	1
Other	0.042			
Labor market status				
Permanent work contract	0.033		0	1
Temporary work contract	0.022		0	1
Participating in education	0.068		0	1
Job-seeking	0.173		0	1
Inactive	0.704		0	1
German language proficiency				
None at all	0.103		0	1
Not very good	0.378		0	1
Average	0.351		0	1
Good	0.149		0	1
Very good	0.018		0	1
Missing information	0.001		0	1
Location of partner				
No partner	0.317		0	1
In Germany	0.591		0	1

Abroad	0.084	0	1
Missing information	0.008	0	1
Type of residence			
Shared accommodation/other	0.328	0	1
Private flat/house	0.668	0	1
Missing information	0.005	0	1
Third person present at interview			
Partner	0.251	0	1
Other third person	0.264	0	1
Nobody	0.436	0	1
Partner and other third person	0.049	0	1
Federal state			
Schleswig-Holstein	0.051	0	1
Hamburg	0.023	0	1
Lower Saxony	0.101	0	1
Bremen	0.013	0	1
North Rhine-Westphalia	0.212	0	1
Hesse	0.099	0	1
Rhineland-Palatinate	0.041	0	1
Baden-Wuerttemberg	0.126	0	1
Bavaria	0.131	0	1
Saarland	0.024	0	1
Berlin	0.042	0	1
Brandenburg	0.033	0	1
Mecklenburg-Hither Pomerania	0.015	0	1
Saxony	0.033	0	1
Saxony-Anhalt	0.024	0	1
Thuringia	0.029	0	1
Missing information	0.005	0	1
Wave			
Wave 1	0.600	0	1
Wave 2	0.400	0	1
N	3957		

Note. 1) if non-missing information on mode; 2) if non-missing information on financing; 3) if non-missing information on events; 4) if responsed to sensitive questions.

Data: IAB-BAMF-SOEP Survey of Refugees, waves 1 to 4.

Table S3 Descriptive statistics of time-varying postarrival variables by individual survey participation (number of waves)

	1	2	3	4	Total
Legal status					
In process	23.60	10.95	9.12	4.94	14.39
Protection granted	68.71	83.10	85.44	89.09	79.17
Protection denied	2.10	2.23	2.74	2.67	2.34
Other status	4.17	2.48	1.08	1.34	2.65
Missing information	1.42	1.24	1.62	1.95	1.45
Labor market status					
Permanent work contract	3.31	9.76	14.72	17.08	9.27
Temporary work contract	2.17	7.98	12.32	14.40	7.50
Participating in education	6.82	10.34	10.31	9.98	9.09
Job-seeking	17.31	19.31	19.51	18.93	18.63
Inactive	70.38	52.61	43.14	39.61	55.50
German language proficiency					
None at all	10.26	2.94	1.62	1.44	5.04
Not very good	37.83	22.86	16.92	12.14	25.78
Average	35.13	38.22	40.02	40.02	37.71
Good	14.91	29.30	31.48	37.96	25.56
Very good	1.77	6.59	9.97	8.44	5.84
Missing information	0.10	0.10	0.00	0.00	0.07
Type of residence					
Shared accommodation/other	32.75	17.38	12.55	9.67	20.94
Private flat/house	66.79	82.26	86.91	90.33	78.65
Missing information	0.45	0.35	0.54	0.00	0.40
N	3957	3946	2589	972	11464

Data: IAB-BAMF-SOEP Survey of Refugees, waves 1 to 4.

Table S4 Descriptive statistics of the controls by individual survey participation (number of waves)

	1	2	3	4	Total
Third person present at interview					
Partner	25.09	17.61	17.27	13.68	19.78
Other third person	26.38	18.04	12.63	8.44	18.89
Nobody	43.64	53.37	62.77	73.56	53.85
Partner and other third person	4.88	10.97	7.34	4.32	7.48
Federal State					
Schleswig-Holstein	5.13	5.04	4.98	5.66	5.11
Hamburg	2.30	2.38	2.09	2.26	2.28
Lower Saxony	10.06	10.29	9.46	8.13	9.84
Bremen	1.29	1.29	1.62	1.23	1.36
North Rhine-Westphalia	21.15	21.92	21.86	19.03	21.40
Hesse	9.93	10.09	10.27	8.44	9.94
Rhineland-Palatinate	4.09	4.08	4.13	2.67	3.98
Baden-Wuerttemberg	12.56	12.44	12.67	16.67	12.89
Bavaria	13.07	12.80	12.21	12.45	12.73
Saarland	2.40	2.26	2.36	3.40	2.42
Berlin	4.22	4.21	4.02	3.91	4.14
Brandenburg	3.31	3.22	3.09	4.01	3.29
Mecklenburg-Hither Pomerania	1.47	1.32	1.31	0.82	1.33
Saxony	3.29	3.27	3.51	4.63	3.45
Saxony-Anhalt	2.43	2.33	2.97	4.22	2.67
Thuringia	2.86	2.71	2.90	2.47	2.78
Missing information	0.45	0.35	0.54	0.00	0.40
Survey year					
2016	59.97	-	-	-	20.70
2017	40.03	49.32	-	-	30.79
2018	-	41.41	51.37	-	25.85
2019	-	9.28	48.63	100.00	22.65
N	3957	3946	2589	972	11464

Data: IAB-BAMF-SOEP Survey of Refugees, waves 1 to 4.

Table S5 Associations between prearrival experiences and life satisfaction and self-rated health at the first interview: Omitted controls

**Panel A: LIFE SATISFACTION**					
	Model 1.3	Model 1.4	Model 1.5	Model 1.6	Model 1.7
Country of origin (ref. Syria)					
Afghanistan	0.387**	0.423***	0.404***	0.429***	0.425***
Eritrea	0.606***	0.615***	0.617***	0.684***	0.712***
Iraq	0.225*	0.240*	0.241*	0.238*	0.217*
Survey wave at first interview (ref. 2016)					
2017	-0.033	-0.01	-0.034	-0.036	-0.017
Age	-0.059*	-0.065*	-0.064*	-0.063*	-0.059*
Age squared	0.001+	0.001*	0.001+	0.001+	0.001+
Legal status (ref. In process)					
Protection granted	0.407***	0.394***	0.400***	0.373***	0.377***
Protection denied	0.077	0.053	0.068	0.112	0.115
Other status	0.285	0.266	0.291	0.257	0.234
Missing information	-0.424	-0.455	-0.442	-0.477	-0.499
Arrival year (ref. 2016)					
2014	-0.269+	-0.296*	-0.266+	-0.233	-0.262+
2015	-0.116	-0.103	-0.115	-0.092	-0.083
Third person present at interview (ref. Partner)					
Other third person	-0.349**	-0.359**	-0.362**	-0.364**	-0.354**
Nobody	-0.258*	-0.268**	-0.264**	-0.262**	-0.256*
Partner and other third person	-0.351+	-0.339+	-0.361+	-0.358+	-0.351+
Gender (ref. Female)					
Male	-0.09	-0.089	-0.094	-0.085	-0.067
Type of residence (ref. Shared accommodation)					
Private flat/house	0.726***	0.722***	0.726***	0.723***	0.715***
Missing information	0.000	0.000	0.000	0.000	0.000
Highest professional educational attainment (ref. No professional education)					
Vocational education	-0.245	-0.237	-0.234	-0.228	-0.238
University	-0.558***	-0.545***	-0.551***	-0.549***	-0.545***
Other	0.040	0.074	0.041	0.029	0.023
Missing information	-0.448	-0.552	-0.420	-0.408	-0.548
German language proficiency (ref. None at all)					
Not very good	0.105	0.108	0.098	0.118	0.127
Average	0.445***	0.444***	0.432**	0.461***	0.474***
Good	0.399*	0.390*	0.382*	0.409**	0.416**
Very good	0.915**	0.934**	0.922**	0.935**	0.907**
Missing information	0.711	0.679	0.711	0.63	0.661
Socioeconomic status before migration (ref. Low)					
Medium	0.148	0.164+	0.157+	0.147	0.149
High	-0.047	-0.018	-0.03	-0.021	-0.029
Missing information	0.625	0.716+	0.669+	0.665+	0.643+
Residence of partner (ref. No partner)					
In Germany	0.435***	0.428***	0.434***	0.429***	0.427***
Abroad	-0.266+	-0.267+	-0.277+	-0.258+	-0.247
Missing information	0.269	0.287	0.307	0.258	0.212
Dummy for answering sensitive questions	0.435***	0.428***	0.434***	0.429***	0.427***
Constant	7.805***	7.515***	7.545***	7.534***	7.813***
Number of persons	3957	3957	3957	3957	3957
Adj. R^2	0.069	0.07	0.068	0.073	0.074
F Statistic	6.546	6.823	6.551	7.097	6.252

**Panel B: SELF-RATED HEALTH**					
	Model 2.3	Model 2.4	Model 2.5	Model 2.6	Model 2.7
Country of origin (ref. Syria)					
Afghanistan	-0.132*	-0.119*	-0.124*	-0.107*	-0.115*
Eritrea	0.227**	0.225**	0.232**	0.277***	0.295***
Iraq	-0.087+	-0.075	-0.079	-0.08	-0.093+

Survey wave at first interview (ref. 2016)					
2017	-0.058	-0.053	-0.059	-0.060	-0.060
Age	0.002	0.000	-0.001	0.001	0.002
Age squared	-0.000*	-0.000*	-0.000*	-0.000*	-0.000**
Legal status (ref. In process)					
Protection granted	0.093*	0.091*	0.091*	0.074+	0.074+
Protection denied	0.008	0.004	0.003	0.037	0.037
Other	0.001	-0.002	0.002	-0.021	-0.022
Missing information	-0.064	-0.065	-0.062	-0.095	-0.093
Arrival year (ref. 2016)					
2014	-0.097	-0.099	-0.09	-0.074	-0.070
2015	0.029	0.030	0.030	0.044	0.048
Third person present at interview (ref. Partner)					
Other third person	-0.117*	-0.121*	-0.122*	-0.123*	-0.123*
Nobody	-0.158***	-0.160***	-0.159***	-0.158***	-0.155***
Partner and other third person	-0.115	-0.111	-0.118	-0.117	-0.121
Gender (ref. Female)					
Male	0.293***	0.293***	0.291***	0.300***	0.302***
Type of residence (ref. Shared accommodation)					
Private flat/house	0.055	0.054	0.055	0.051	0.051
Missing information	0.000	0.000	0.000	0.000	0.000
Highest professional educational attainment (ref. No professional education)					
Vocational education	0.023	0.028	0.026	0.033	0.025
University	0.036	0.04	0.037	0.042	0.037
Other	0.165	0.175	0.166	0.156	0.145
Missing information	-0.844***	-0.907***	-0.836***	-0.824***	-0.898***
German language proficiency (ref. None at all)					
Not very good	0.101+	0.100+	0.096+	0.112*	0.108+
Average	0.231***	0.228***	0.223***	0.246***	0.244***
Good	0.376***	0.369***	0.365***	0.385***	0.385***
Very good	0.562***	0.566***	0.572***	0.569***	0.566***
Missing information	0.151	0.158	0.175	0.114	0.127
Socioeconomic status before migration (ref. Low)					
Medium	0.104*	0.112**	0.107*	0.103*	0.098*
High	0.112*	0.125**	0.118*	0.127**	0.117*
Missing information	0.345*	0.367*	0.359*	0.347*	0.351*
Residence of partner (ref. No partner)					
In Germany	0.037	0.036	0.034	0.035	0.031
Abroad	-0.028	-0.031	-0.033	-0.021	-0.016
Missing information	-0.193	-0.181	-0.183	-0.209	-0.23
Dummy for answering sensitive questions	0.054	0.114+	0.103	0.169***	0.148+
Constant	4.077***	3.994***	4.018***	3.999***	4.096***
Number of persons	3957	3957	3957	3957	3957
Adj. R^2	0.131	0.131	0.131	0.14	0.141
F Statistic	12.281	12.681	12.485	13.654	11.798

Note. Statistical significance at: + $p < 0.10$, * $p < 0.05$, ** $p < 0.01$, *** $p < 0.001$. OLS regression coefficients. Dependent variables: life satisfaction (0 to 10) and self-rated health (1 to 5). Further controls not shown: federal state fixed effects.

Data: IAB-BAMF-SOEP Survey of Refugees, waves 1 to 4.

Table S6 Associations between postarrival experiences and life satisfaction and self-rated health: Omitted controls

**Panel A: LIFE SATISFACTION**	Model 3.1	Model 3.2	Model 3.3	Model 3.4	Model 3.5
Wave dummy (ref. wave 1)					
Wave 2	0.336**	0.336**	0.316**	0.338**	0.275*
Wave 3	0.486*	0.466*	0.440*	0.465*	0.370+
Wave 4	0.588*	0.553*	0.512+	0.552*	0.411
Age	0.015	0.006	0.004	-0.001	-0.024
Age squared	0.000	0.000	0.000	0.000	0.001
Federal state (ref. Schleswig-Holstein)					
Hamburg	0.558	0.523	0.584	0.704	0.657
Lower Saxony	0.235	0.222	0.204	0.256	0.21
Bremen	0.538	0.598	0.448	0.424	0.373
North Rhine-Westphalia	-0.455	-0.458	-0.469	-0.403	-0.417
Hesse	-0.541	-0.532	-0.534	-0.457	-0.44
Rhineland-Palatinate	-2.104	-2.083	-2.068	-2.092	-2.037
Baden-Wuerttemberg	-0.541	-0.569	-0.567	-0.409	-0.387
Bavaria	0.593	0.558	0.552	0.71	0.663
Saarland	-1.135+	-1.107+	-1.117+	-1.182*	-1.162*
Berlin	0.028	-0.064	0.005	0.186	0.149
Brandenburg	1.538*	1.540*	1.553*	1.676*	1.597*
Mecklenburg-Hither Pomerania	0.409	0.373	0.33	0.436	0.496
Saxony	0.701	0.653	0.595	0.687	0.560
Saxony-Anhalt	0.619	0.625	0.598	0.606	0.608
Thuringia	-0.593	-0.63	-0.649	-0.63	-0.681
Missing information	-0.392	-0.404	-0.427	0.083	0.056
Third person present at interview (ref. Partner)					
Other third person	-0.155*	-0.152*	-0.151*	-0.155*	-0.148*
Nobody	-0.255***	-0.252***	-0.249***	-0.247***	-0.243***
Partner and other third person	-0.184*	-0.188*	-0.192*	-0.188*	-0.185*
Panel Conditioning (ref. first interview)					
2nd interview	-0.367**	-0.361**	-0.393***	-0.414***	-0.443***
3rd interview	-0.544**	-0.540**	-0.573**	-0.600***	-0.625***
4th interview	-0.652**	-0.645**	-0.675**	-0.708**	-0.719**
Constant	7.029***	7.202***	7.069***	6.918***	7.110***
Person-years	11464	11464	11464	11464	11464
Number of persons	3957	3957	3957	3957	3957
R^2 overall	0.002	0.002	0.002	0.006	0.009
R^2 within	0.009	0.01	0.01	0.014	0.018

**Panel B: SELF-RATED HEALTH**	Model 4.1	Model 4.2	Model 4.3	Model 4.4	Model 4.5
Wave dummy (ref. wave 1)					
Wave 2	0.086	0.088+	0.081	0.096+	0.061
Wave 3	0.186*	0.179*	0.174+	0.197*	0.141
Wave 4	0.142	0.129	0.117	0.152	0.077
Age	0.071*	0.068*	0.067*	0.072*	0.057+
Age squared	-0.001**	-0.001**	-0.001*	-0.001**	-0.001*
Federal state (ref. Schleswig-Holstein)					
Hamburg	0.430+	0.418+	0.434+	0.439+	0.437+
Lower Saxony	0.179	0.186	0.168	0.182	0.174
Bremen	0.034	0.044	0.003	0.036	-0.018
North Rhine-Westphalia	0.029	0.036	0.026	0.035	0.033
Hesse	0.451+	0.460+	0.458+	0.462+	0.466+
Rhineland-Palatinate	-0.452	-0.424	-0.432	-0.448	-0.412
Baden-Wuerttemberg	-0.03	-0.034	-0.035	-0.023	-0.014
Bavaria	0.033	0.036	0.025	0.049	0.027
Saarland	-0.177	-0.168	-0.159	-0.169	-0.179
Berlin	0.249	0.232	0.258	0.27	0.244
Brandenburg	0.38	0.371	0.379	0.394	0.368

Mecklenburg-Hither Pomerania	0.200	0.230	0.199	0.213	0.223
Saxony	-0.706	-0.706	-0.743	-0.701	-0.754
Saxony-Anhalt	0.033	0.047	0.034	0.037	0.036
Thuringia	-0.133	-0.148	-0.159	-0.142	-0.161
Missing information	-0.053	-0.054	-0.06	-0.008	-0.03
Third person present at interview (ref. Partner)					
Other third person	-0.014	-0.014	-0.012	-0.014	-0.012
Nobody	-0.036	-0.034	-0.031	-0.033	-0.034
Partner and other third person	0.065	0.062	0.061	0.062	0.064
Panel Conditioning (ref. first interview)					
2nd interview	-0.092+	-0.094+	-0.104*	-0.096+	-0.110*
3rd interview	-0.149+	-0.152+	-0.162+	-0.154+	-0.168+
4th interview	-0.164	-0.164	-0.174	-0.168	-0.178
Constant	2.898***	2.950***	2.917***	2.862***	3.010***
Person-years	11464	11464	11464	11464	11464
Number of persons	3957	3957	3957	3957	3957
R^2 overall	0.029	0.03	0.032	0.023	0.044
R^2 within	0.009	0.009	0.01	0.008	0.012

Note. Statistical significance at: + $p < 0.10$, * $p < 0.05$, ** $p < 0.01$, *** $p < 0.001$. FE regression coefficients. Dependent variables: Overall life satisfaction (0 to 10) and self-rated health (1 to 5).

Data: IAB-BAMF-SOEP Survey of Refugees, waves 1 to 4.

Table S7 Life satisfaction and postarrival experiences over time

**LIFE SATISFACTION**	Model S7.1	Model S7.2	Model S7.3	Model S7.4	Model S7.5
Labor market status (ref. Never received a permanent work contract)					
Permanent work contract at first interview	0.214+	0.257			
Transition to permanent work contract	0.149*	0.125			
Legal status (ref. Never granted protection *status)*					
Granted protection at first interview	0.839***		0.833***		
Transition to granted protection	0.673***		0.479**		
Language proficiency (ref. Never good proficiency)					
Good proficiency at first interview	0.181*			0.283**	
Transition to good proficiency	0.118*			0.141+	
Accommodation (ref. Never in private flat)					
In private flat at first interview	0.947***				1.105***
Transition to private flat	0.622***				0.363*
Years since first interview (ref. 0)					
1 year since first interview	-0.122	-0.166*	-0.441*	-0.133	-0.370*
2 years since first interview	-0.05	-0.095	-0.368	-0.063	-0.340
3 years since first interview	0.053	-0.053	-0.164	-0.082	-0.199
Interactions of mechanisms and time dummies					
1 year # Permanent work contract at first interview		-0.149			
1 year # Transition to permanent work contract		0.089			
2 years # Permanent work contract at first interview		-0.104			
2 years # Transition to permanent work contract		0.005			
3 years # Permanent work contract at first interview		0.144			
3 years # Transition to permanent work contract		0.128			
1 year # Granted protection at first interview			0.394*		
1 year # Transition to granted protection			0.391*		
2 years # Granted protection at first interview			0.359+		
2 years # Transition to granted protection			0.601**		
3 years # Granted protection at first interview			0.207		
3 years # Transition to granted protection			0.558*		
1 year # Good proficiency at first interview				-0.125	
1 year # Transition to good proficiency				0.019	
2 years # Good proficiency at first interview				0.023	
2 years # Transition to good proficiency				-0.064	
3 years # Good proficiency at first interview				0.227	
3 years # Transition to good proficiency				0.141	
1 year # In private flat at first interview					0.096
1 year # Transition to private flat					0.680***
2 years # In private flat at first interview					0.096
2 years # Transition to private flat					0.666***
3 years # In private flat at first interview					-0.032
3 years # Transition to private flat					0.714**
**Controls**					
Country of origin (ref. Syria)					
Afghanistan	0.427***	-0.025	0.295***	-0.009	0.206**
Eritrea	0.412***	0.160	0.270**	0.202+	0.413***
Iraq	0.244**	-0.000	0.209**	0.005	0.071
Wave dummy (ref. wave 1)					
Wave 2	0.142+	0.175*	0.105	0.161*	0.170*
Wave 3	0.070	0.129	-0.003	0.109	0.142
Wave 4	0.003	0.092	-0.057	0.052	0.112
Age	0.011	0.019	0.016	0.028	0.004
Age squared	-0.000	-0.000	-0.000	-0.000	-0.000
Federal state (ref. Schleswig-Holstein)					
Hamburg	0.129	-0.078	-0.136	-0.046	0.230
Lower Saxony	0.026	0.116	0.092	0.112	0.063
Bremen	-0.328+	-0.109	-0.273	-0.062	-0.241

North Rhine-Westphalia	-0.005	0.050	-0.015	0.079	0.050
Hesse	-0.064	-0.128	-0.162	-0.082	-0.006
Rhineland-Palatinate	-0.462**	-0.407*	-0.472**	-0.370*	-0.416*
Baden-Wuerttemberg	0.117	0.040	0.022	0.064	0.171
Bavaria	0.181	0.148	0.086	0.171	0.271*
Saarland	-0.056	0.103	0.016	0.133	0.053
Berlin	-0.249	-0.462**	-0.542**	-0.433*	-0.158
Brandenburg	-0.179	-0.202	-0.272	-0.186	-0.154
Mecklenburg-Hither Pomerania	-0.054	0.100	-0.001	0.113	-0.003
Saxony	0.044	0.146	0.034	0.161	0.129
Saxony-Anhalt	0.248	0.448**	0.329*	0.426**	0.319*
Thuringia	-0.459**	-0.323+	-0.410*	-0.313+	-0.404*
Missing information	-0.191	-0.037	-0.109	-0.002	0.075
Socioeconomic status before migration (ref. Low)					
Medium	0.084	0.137*	0.111+	0.134*	0.107
High	-0.056	0.023	-0.023	0.007	-0.009
Missing information	0.002	-0.018	0.036	-0.012	0.013
Arrival year (ref. 2016)					
2014	0.093	0.318***	0.275**	0.274**	0.154
2015	0.016	0.098	0.075	0.079	0.031
Third person present at interview (ref. Partner)					
Other third person	-0.289***	-0.328***	-0.316***	-0.334***	-0.286***
Nobody	-0.359***	-0.387***	-0.380***	-0.393***	-0.337***
Partner and other third person	-0.132	-0.168*	-0.151+	-0.179*	-0.146+
Gender (ref. Female)					
Male	-0.162**	-0.224***	-0.166**	-0.217***	-0.106*
Highest professional educational attainment (ref. No professional education)					
Vocational education	-0.082	-0.029	-0.058	-0.043	-0.027
University	-0.431***	-0.348***	-0.368***	-0.401***	-0.365***
Other	0.141	0.105	0.160	0.121	0.166
Missing information	-0.007	-0.169	-0.161	-0.173	0.018
Dummy for answering sensitive questions	0.051	0.065	0.060	0.061	0.073
Ever missing legal status	0.046		0.103		
Ever missing language proficiency	0.165			0.044	
Ever missing accommodation	0.271				0.262
Constant	5.787***	7.218***	6.636***	6.961***	6.587***
Person-years	11464	11464	11464	11464	11464
Number of persons	3957	3957	3957	3957	3957
R^2 overall	0.059	0.028	0.045	0.029	0.051
R^2 within	0.004	0.005	0.007	0.005	0.012

Note. Statistical significance at: + $p < 0.10$, * $p < 0.05$, ** $p < 0.01$, *** $p < 0.001$. REGC regression coefficients. Figures 1 and 2 are based on these regressions. Dependent variables: overall life satisfaction (0 to 10).

Data: IAB-BAMF-SOEP Survey of Refugees, waves 1 to 4.

Table S8 Self-rated health and postarrival experiences over time

**SELF-RATED HEALTH**	Model S8.1	Model S8.2	Model S8.3	Model S8.4	Model S8.5
Labor market status (ref. Never received permanent work contract)					
Permanent work contract at first interview	0.054	0.128			
Transition to permanent work contract	0.174***	0.175***			
Legal status (ref. Never granted protection status)					
Granted protection at first interview	0.045		0.116+		
Transition to granted protection	-0.016		-0.025		
Language proficiency (ref. Never good proficiency)					
Good proficiency at first interview	0.247***			0.313***	
Transition to good proficiency	0.188***			0.161***	
Accommodation (ref. Never in private flat)					
In private flat at first interview	0.175***				0.238***
Transition to private flat	0.128*				0.154*
Years since first interview (ref. 0)					
1 year since first interview	0.058	0.049	0.088	0.048	0.083
2 years since first interview	0.155*	0.128*	0.220*	0.124+	0.152
3 years since first interview	0.266**	0.217*	0.313*	0.205*	0.138
Interactions of mechanisms and time dummies					
1 year # Permanent work contract at first interview		-0.097			
1 year # Transition to permanent work contract		-0.001			
2 years # Permanent work contract at first interview		-0.111			
2 years # Transition to permanent work contract		0.044			
3 years # Permanent work contract at first interview		-0.030			
3 years # Transition to permanent work contract		0.063			
1 year # Granted protection at first interview			-0.034		
1 year # Transition to granted protection			0.035		
2 years # Granted protection at first interview			-0.065		
2 years # Transition to granted protection			0.029		
3 years # Granted protection at first interview			-0.050		
3 years # Transition to granted protection			0.079		
1 year # Good proficiency at first interview				-0.104*	
1 year # Transition to good proficiency				0.065	
2 years # Good proficiency at first interview				-0.049	
2 years # Transition to good proficiency				0.066	
3 years # Good proficiency at first interview				-0.006	
3 years # Transition to good proficiency				0.096+	
1 year # In private flat at first interview					-0.054
1 year # Transition to private flat					-0.006
2 years # In private flat at first interview					-0.040
2 years # Transition to private flat					0.031
3 years # In private flat at first interview					0.093
3 years # Transition to private flat					0.125
**Controls**					
Country of origin (ref. Syria)					
Afghanistan	-0.059	-0.136***	-0.097*	-0.124***	-0.096*
Eritrea	0.227***	0.172***	0.223***	0.212***	0.250***
Iraq	-0.081*	-0.118**	-0.098**	-0.112**	-0.108**
Wave dummy (ref. wave 1)					
Wave 2	-0.018	-0.000	-0.032	-0.017	-0.000
Wave 3	-0.040	-0.008	-0.059	-0.025	-0.007
Wave 4	-0.200*	-0.150+	-0.220*	-0.175*	-0.151+
Age	0.017+	0.009	0.010	0.021*	0.008
Age squared	-0.001***	-0.001***	-0.001***	-0.001***	-0.001***
Federal state (ref. Schleswig-Holstein)					
Hamburg	0.308***	0.247**	0.238**	0.280***	0.305***
Lower Saxony	0.200**	0.223***	0.226***	0.211**	0.215**
Bremen	0.173	0.164	0.144	0.211+	0.141

North Rhine-Westphalia	0.030	0.014	0.012	0.044	0.016
Hesse	0.204**	0.164*	0.174**	0.213**	0.194**
Rhineland-Palatinate	-0.059	-0.080	-0.089	-0.048	-0.081
Baden-Wuerttemberg	-0.007	-0.034	-0.022	-0.014	-0.003
Bavaria	0.082	0.062	0.069	0.082	0.093
Saarland	-0.097	-0.075	-0.073	-0.058	-0.070
Berlin	-0.080	-0.154+	-0.154+	-0.122	-0.098
Brandenburg	0.135	0.112	0.092	0.127	0.116
Mecklenburg-Hither Pomerania	0.090	0.100	0.078	0.107	0.070
Saxony	-0.065	-0.077	-0.090	-0.061	-0.082
Saxony-Anhalt	0.014	0.056	0.024	0.033	0.012
Thuringia	-0.142+	-0.123	-0.129	-0.118	-0.132
Missing information	-0.124	-0.079	-0.080	-0.062	-0.112
Socioeconomic status before migration (ref. Low)					
Medium	0.109***	0.119***	0.118***	0.115***	0.114***
High	0.098**	0.123***	0.117**	0.104**	0.116**
Missing information	0.150	0.164	0.167	0.155	0.173
Arrival year (ref. 2016)					
2014	-0.054	0.033	0.021	-0.015	0.010
2015	-0.017	0.018	0.009	-0.003	0.006
Third person present at interview (ref. Partner)					
Other third person	-0.060*	-0.059*	-0.060*	-0.064*	-0.051+
Nobody	-0.067**	-0.064**	-0.061*	-0.072**	-0.052*
Partner and other third person	0.031	0.029	0.028	0.021	0.032
Gender (ref. female)					
Male	0.248***	0.266***	0.306***	0.268***	0.321***
Highest professional educational attainment (ref. No professional education)					
Vocational education	0.021	0.045	0.051	0.029	0.052
University	0.037	0.103**	0.107**	0.043	0.105**
Other	0.139	0.159+	0.192*	0.166	0.194+
Missing information	-0.385+	-0.411+	-0.398+	-0.409+	-0.359
Dummy for answering sensitive questions	0.020	0.031	0.036	0.026	0.037
Ever missing legal status	-0.054		-0.036		
Ever missing language proficiency	-0.087			-0.113	
Ever missing accommodation	0.123				0.147
Constant	3.678***	4.063***	4.003***	3.782***	3.879***
Person-years	11464	11464	11464	11464	11464
Number of persons	3957	3957	3957	3957	3957
R^2 overall	0.148	0.136	0.133	0.142	0.135
R^2 within	0.006	0.006	0.006	0.007	0.006

Note. Statistical significance at: + p < 0.10, * p < 0.05, ** p < 0.01, *** p < 0.001. REGC regression coefficients. Figures 1 and 3 are based on these regressions. Dependent variables: self-rated health (1 to 5).

Data: IAB-BAMF-SOEP Survey of Refugees, waves 1 to 4.
